# GRAS gene family in rye (*Secale cereale* L.): genome-wide identification, phylogeny, evolutionary expansion and expression analyses

**DOI:** 10.1186/s12870-023-04674-1

**Published:** 2024-01-13

**Authors:** Yu Fan, Xianqi Wan, Xin Zhang, Jieyu Zhang, Chunyu Zheng, Qiaohui Yang, Li Yang, Xiaolong Li, Liang Feng, Liang Zou, Dabing Xiang

**Affiliations:** 1 Key Laboratory of Coarse Cereal Processing, Ministry of Agriculture and Rural Affairs, Sichuan Engineering & Technology Research Center of Coarse Cereal Industralization, College of Food and Biological engineering, Chengdu University, Longquanyi District, Chengdu, 610106 Sichuan Province P.R. China; 2 Sichuan Academy of Agricultural Machinery Science, Chengdu, 610011 P.R. China; 3 College of Food Science and Engineering, Xinjiang Institute of Technology, Aksu, 843100 P.R. China; 4 Chengdu Institute of Food Inspection, Chengdu, 610000 P.R. China

**Keywords:** *Secale cereale*, *GRAS* gene family, Expression pattern, DELLA

## Abstract

**Background:**

The *GRAS* transcription factor family plays a crucial role in various biological processes in different plants, such as tissue development, fruit maturation, and environmental stress. However, the GRAS family in rye has not been systematically analyzed yet.

**Results:**

In this study, 67 *GRAS* genes in *S. cereale* were identified and named based on the chromosomal location. The gene structures, conserved motifs, cis-acting elements, gene replications, and expression patterns were further analyzed. These 67 *ScGRAS* members are divided into 13 subfamilies. All members include the LHR I, VHIID, LHR II, PFYRE, and SAW domains, and some nonpolar hydrophobic amino acid residues may undergo cross-substitution in the VHIID region. Interested, tandem duplications may have a more important contribution, which distinguishes them from other monocotyledonous plants. To further investigate the evolutionary relationship of the *GRAS* family, we constructed six comparative genomic maps of homologous genes between rye and different representative monocotyledonous and dicotyledonous plants. The response characteristics of 19 *ScGRAS* members from different subfamilies to different tissues, grains at filling stages, and different abiotic stresses of rye were systematically analyzed. Paclobutrazol, a triazole-based plant growth regulator, controls plant tissue and grain development by inhibiting gibberellic acid (GA) biosynthesis through the regulation of DELLA proteins. Exogenous spraying of paclobutrazol significantly reduced the plant height but was beneficial for increasing the weight of 1000 grains of rye. Treatment with paclobutrazol, significantly reduced gibberellin levels in grain in the filling period, caused significant alteration in the expression of the DELLA subfamily gene members. Furthermore, our findings with respect to genes, *ScGRAS46* and *ScGRAS60*, suggest that these two family members could be further used for functional characterization studies in basic research and in breeding programmes for crop improvement.

**Conclusions:**

We identified 67 ScGRAS genes in rye and further analysed the evolution and expression patterns of the encoded proteins. This study will be helpful for further analysing the functional characteristics of *ScGRAS* genes.

**Supplementary Information:**

The online version contains supplementary material available at 10.1186/s12870-023-04674-1.

## Introduction

Transcription factors are a class of DNA-binding proteins that regulate gene transcription by binding specifically to cis-acting elements in the promoter region of eukaryotic genes, through interactions with each other proteins [1]. *GRAS* gene family is present only in higher plants [2]. Its members have unique GRAS domains, and some of them also have DELLA protein structures. These domains are closely related to physiological processes such as plant growth, metabolism, and stress adaptation [2]. GRAS protein is named after the characteristic letters of the three members initially discovered: *GAI* (GIBBERELLIN INSENSITIVE) [3], *RGA* (REPRESSOR OF GAL-3) [4], and *SCR* (SCARECROW) [5]. The members of the GRAS protein family generally consist of 400 to 700 amino acid residues. The length and sequence of amino acids are highly complex due to their N-terminal structures, while the C-terminal amino acids are relatively conserved [6]. In general, the typical structural domains of the GRAS family include LHR I (Leucine heptad repeat I), VHIID (Val-His-Ile-Ile-Asp), LHR II, PFYRE (Pro-Phe-Tyr-Arg-Glu), and SAW (Ser-Ala-Trp) [7]. VHIID is considered the core region as it is highly conserved. It binds with two leucine heptad repeat regions to form LHR I - VHIID - LHR II complexes, regulating the binding activity with DNA and other proteins [6, 8]. Moreover, these two leucine-rich regions are composed of about 100 amino acid residues. In most cases, these two regions do not form a complete unit every seven residues, which distinguishes them from the Leucine zipper [9]. There is a hypothetical nuclear localization signal in the LHR I region at the C - terminus, therefore the SV40-type sequence could be recognized [10, 11]. Some LHR I motifs in the N-terminal of GRAS proteins contain a conserved LXXLL sequence (Leu-X-X-Leu, X represents any amino acid), which is common in most GRAS proteins [12–15]. However, the roles of the LXXLL sequences in mediating the interactions of plant GRAS proteins with their regulators and co-activators are still unclear. PFYRE motif, which contains a tyrosine phosphorylation site, is not as conserved as the VHIID region, but still exhibits high similarity and collinearity in most GRAS proteins. This motif typically consists of three parts: proline residue (P), phenylalanine residue (F), tyrosine residue (Y), arginine residue (R), and glutamate residue (E) [6, 11]. The functions of the PFYRE and SAW motifs are not fully understood yet, but they both have conserved amino acid residues or pairs, suggesting that these two structural motifs are important for protein function or stability [16]. N-terminal region of GRAS proteins is flexible and variable in length and sequence, forming inherently disordered regions (IDRs) that adopt specific molecular recognition features upon binding [9]. The diverse N-terminal interacts with different target proteins during expression, acting cooperatively and exhibiting protein specificity, which plays a key role in signal transduction pathways, depending on the different members or expression conditions [9, 16, 17].

According to the members of the GRAS family in the genomes of *Arabidopsis* and rice, this family can be divided into eight branches, including SCL3 (SCARECROW - LIKE3), SHR (SHORT ROOT), PAT1 (PHYTOCHROME A SIGNAL TRANSACTION), LISCL (*Lilium longiflorum* SCR like), DELLA, SCR (GAI - RGA - SCR), LAS (LATERAL SUPPRESSOR), and HAM (HAIRY MERISTEM) [18]. These subfamilies play their respective roles in plant growth, development, and metabolic regulation. Cenci and Rouard [7] also analyzed the GRAS transcription factors in various angiosperms, who found that there were other subfamilies such as DLT (Dwarf and Low Tillering, NSP1 (Nodulation Signaling Pathway 1), NSP2 besides the above eight subfamilies. Currently, the GRAS family has been reported to exist in over 50 plants, including *Arabidopsis thaliana* (n = 33) [19], *Brachypodium distachyon* (n = 48) [20], *Brassica napus* (n = 92) [21], *Capsicum annuum* (n = 50) [22], *Chenopodium quinoa* (n = 52) [23], *Citrullus lanatus* (n = 37) [24], *Citrus sinensis* (n = 50) [25], *Fagopyrum tataricum* (n = 47) [26], *Glycine max* (n = 117) [27], *Hordeum vulgare* (n = 62) [28], *Jatropha curcas* (n = 48) [29], *Litchi chinensis* (n = 48) [30], *Malus domestica* (n = 127) [31], *Manihot esculenta* (n = 77) [32], *Medicago sativa* (n = 51) [33], *Oryza sativa* (n = 57) [34], *Phaseolus vulgaris* (n = 55) [35], *Ricinus communes* (n = 48) [36], *Setaria italica* (n = 57) [37], *Solanum lycopersicum* (n = 54) [38], *Sorghum bicolor* (n = 81) [39], *Triticum aestivum* (n = 188) [40], *Vitis vinifera* (n = 52) [41], *Zea mays* (n = 86) [42], et al.

The *GRAS* family comprises diverse subfamilies with distinct structural and functional features. Members of different subfamilies may participate in various processes of plant growth, development and environmental adaptation [43–47]. The *SCR* is co-localized with *SHR* in the vascular bundle sheath cells of leaves and roots [48]. *PAT1*, *SCL13*, and *SCL21* are members of the PAT1 subfamily and are implicated in regulating light signal transduction [47, 49]. DELLA is involved in the response to plant hormone signals, such as gibberellin, jasmonic acid, and auxin [50–52]. The protein phosphorylation and dephosphorylation processes that regulate GA signaling in plants are generally mediated by the proteasome-dependent destabilization of DELLA protein repressors, which modulate the response to endogenous gibberellins. Leaf elongation in seedlings that relies on the gibberellin pathway is governed by the proteasome-mediated derepression of DELLA [53]. *LlDELLA1* facilitates flower and pod development in *Lupinus luteus*. Its expression level slightly declines from the flower bud stage to anther opening, but rapidly elevates during pollination, fertilization, podding, and early grain development [54]. *LISCL* is implicated in the meiosis of pollen and facilitates the formation of microspores in *L. longiflorum* [55]. HAM family members from various flowering plants sustain the indeterminacy of shoot meristem and facilitate the formation of re-axillary meristem [45, 56–59]. The loss-of-function of *HAM* leads to a defect in shoot apical meristem in *Capsicum annuum* [58]. *PhHAM* is specifically expressed in the vascular tissue of stem primordia in petunia, which plays a vital role in sustaining the activity of inter shoot apical meristem [59]. In *Arabidopsis*, DELLAs, SCL3, and IDDs constitute a “co-activator/co-repressor exchange regulation system” to fine-tune the feedback regulation of gibberellin [60]. Through the interactions and transcriptional networks among these proteins, they partake in various signaling pathways and physiological events in multiple aspects. DLT, OSH1, and OsOFP19 form functional complexes that play a pivotal role in brassinolide signaling and determining cell division patterns during plant growth and grain development in rice [61]. *OsMOC1* is one of the key factors in determining the number of tillers in rice, which is essential for axillary meristem (AM) formation and bud growth [62]. Furthermore, salt, ultraviolet radiation, flooding, drought, and extreme temperatures can inflict irreversible damage to crop growth and development, ultimately impeding growth and diminishing yield [63]. Some studies have demonstrated that *GRAS* genes play a crucial regulatory role in plant responses to stress. *NtGRAS1* partakes in the phosphorylation process of reactive oxygen species and nitric oxide stress induction in cells, thereby regulating the homeostasis of nutrient distribution within cells [64]. *PeSCL7* is induced by drought and salt stress, which is repressed by gibberellic acid (GA) in poplar. The transgenic *Arabidopsis* plants over-expressing *PeSCL7* exhibited enhanced tolerance to drought and salt treatment due to the increased activity of superoxide dismutase (SOD) and α-amylase (FAA) [65]. Compared with wild-type plants, *OsGRAS23*-overexpressing rice plants showed improved drought resistance and oxidative stress tolerance [66].

Rye (*Secale cereale* L.) is a member of the *Secale* genus in the Poaceae family and contains various nutrients for human consumption, including starch, vitamins, dietary fiber, protein, mineral elements, and phenolic compounds [67]. Rye has multiple applications in food, feed, bioenergy and alcohol production industries [68, 69] and exhibits probiotic activity that can lower the risk of cardiovascular and obesity diseases [70–72]. Rye is also a highly resilient crop that can withstand low temperatures, droughts, and poor soils [73]. As a diploid species in the Triticeae Dumortier, rye is of significant importance and closely related to barley and wheat [74]. Therefore, systematic gene mining and functional characterization of rye are essential for elucidating the physiological functions, evolutionary relationships, and genetic improvement of gramineous crops. In this study, we performed a comprehensive analysis of the ScGRAS family based on the recently published whole genome sequences of rye [75]. 67 GRAS genes were identified in *S. cereale* and assigned them to thirteen subfamilies. Further analysis was conducted on their gene structures, motif compositions, duplications, chromosome distributions, and phylogenetic relationships. We also characterized the expression patterns of selected *ScGRAS* members in different tissues and grain development stages, as well as under different stress and hormone induction. In addition, we investigated the paclobutrazol significantly reduced the plant height of rye, and promoted increase the weight of grains. Paclobutrazol may affect the filling process through the gibberellin pathway in rye.

## Results

### Identification of *GRAS* genes in *S. Cereale*

Based on their position on the rye chromosome, these GRAS members have been renamed *ScGRAS1* to *ScGRAS67* (Table S1). Their basic features including gene coding sequence (CDS), protein molecular weight (MW), isoelectric point (PI), and subcellular localization are systematically analyzed. Of the 67 ScGRAS proteins, ScGRAS57 was the smallest with 395 amino acids. The largest was ScGRAS50 with 1453 amino acids. Molecular weight of the proteins ranged from 41.47 kDa (ScGRAS57) to 163.58 kDa (ScGRAS50). The pI ranged from 4.75 (ScGRAS28) to 10.56 (ScGRAS14), with a median of 5.98. All the putative proteins encoded by the ScGRAS genes, contained the GRAS domain, which is necessary for their function as transcription factors. Based on the predicted subcellular localization, 28 ScGRASs were located in the nucleus, 16 in the chloroplast, 29 in the cytoplasmic, three (*ScGRAS33*, *ScGRAS44*, and *ScGRAS47*) in the mitochondria, two (*ScGRAS35*, and *ScGRAS59*) in the endoplasmic reticulum, two (*ScGRAS3*, and *ScGRAS41*) in the peroxisome (Table S1).

### Phylogenetic analysis, and multiple sequence alignment of ScGRAS putative proteins

**Fig. 1:**
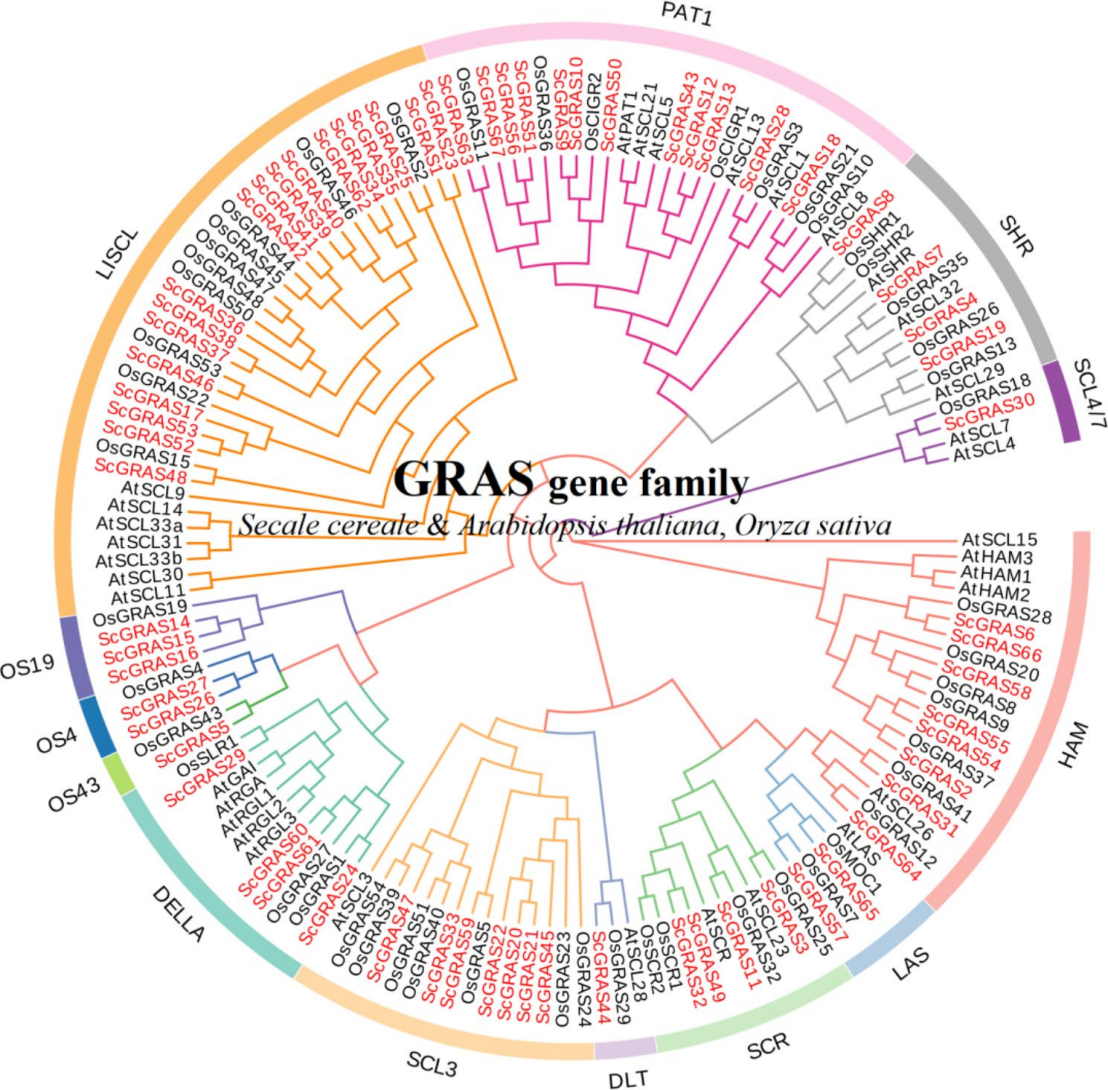
Unrooted phylogenetic tree showing relationships among GRAS domains of *Secale cereale* (Sc), *Arabidopsis thaliana* (At) and *Oryza sativa* (Os). The phylogenetic tree was derived using the neighbor-joining method in MEGA7.0. The tree shows the 13 phylogenetic subfamilies. GRAS proteins from S. cereale are highlighted in red

We constructed a phylogenetic tree encompassing *S. cereale* (67 ScGRASs), *A. thaliana* (33 AtGRASs), and *O. sativa* (46 OsGRASs) through the neighbor-joining method (Fig. 1, Table S1). Following the classification methodology proposed by Cenci et al. [7] and Tian et al. [34], the 146 GRAS proteins were categorized into thirteen distinct topological branches. Notably, LISCL exhibited the largest number of members (18 ScGRAS proteins), while OS43 (ScGRAS5), SCL4/7 (ScGRAS30), and DLT (ScGRAS44) possessed the fewest representatives (solely one ScGRAS protein each). The topology tree reveals a remarkable genetic affinity between certain ScGRAS proteins and numerous AtGRAS and OsGRAS proteins (bootstrap support ≥ 70), exemplified by ScGRAS5, ScGRAS8, and ScGRAS58. This suggests that these homologous proteins may share comparable gene structures and physiological functionalities.

To elucidate the conserved amino acid residues within different subfamilies, a subset of AtGRASs, OsGRASs, and ScGRASs from 13 distinct subfamilies were randomly chosen for comprehensive multi-sequence comparisons (Figure S1, Table S1). The intricate conserved domains, namely LHR I, VHIID, LHR II, PFYRE, and SAW, displayed remarkable complexity among various subfamily members of rye GRAS proteins. The diversity inherent in these amino acids contributes to structural and functional divergences. Notably, the VHIID domain serves as the pivotal region for functionality, exhibiting highly similar amino acid configurations that are readily identifiable across different species. With the exception of GRAS33, GRAS34, GRAS35, and GRAS43, the conservation of His and Asp residues within the VHIID domain remained consistent. Additionally, certain non-polar hydrophobic amino acid residues exhibited potential cross-substitution, hypothesized to have minimal impact on peptide formation. It is worth to named select GRAS members demonstrated alternating residues, oscillating between Ile and Val within the VHLLD region. An observation worth noting is the presence of a highly disordered region at the N-terminus of ScGRAS proteins, showcasing discernible similarities across different subfamilies.

### Gene structures, conserved motifs, and cis-acting elements analysis of *ScGRAS* genes

**Fig. 2:**
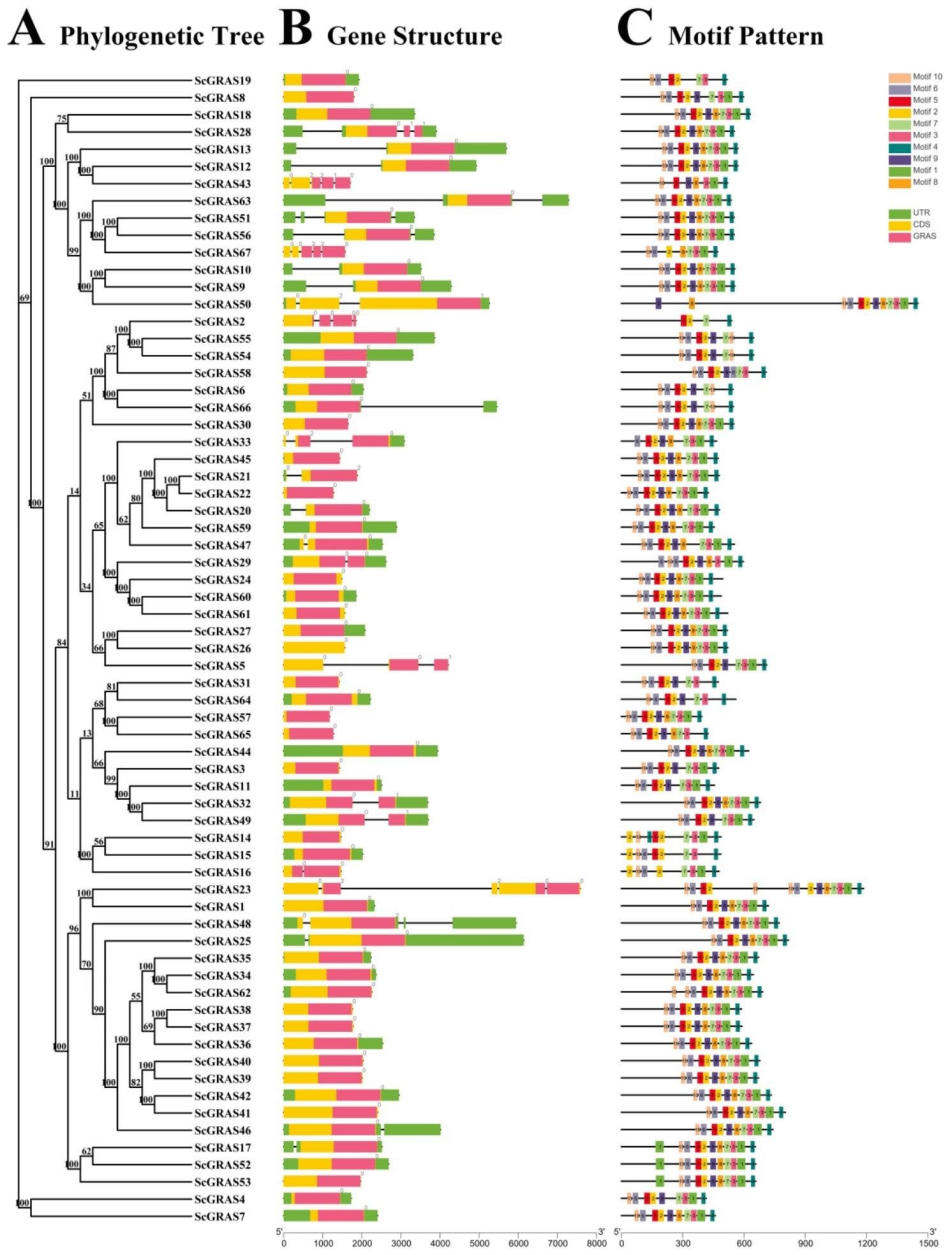
Phylogenetic relationships, gene structure analysis, and motif distributions of *S. cereale GRAS* genes. **A** Phylogenetic tree was constructed using the neighbor-joining method with 1000 replicates for each node. **B** Exons and introns are indicated by yellow rectangles and grey lines, respectively. The green, yellow, and red rectangles represent the UTR, CDS, and GRAS conserved domains, respectively. **C** Amino acid motifs in the *ScGRAS* proteins (1–10) are represented by colored boxes. The black lines indicate relative protein lengths

A comparative analysis of exon-intron structures reveals variations in the number and sequencing among the 67 *ScGRAS* genes, ranging from 1 to 5 exons (Fig. 2A and B, Tables S1). All *ScGRAS* genes contain the GRAS domain, with the majority (40, ~ 59.70%) lacking introns. Fifteen *ScGRAS* genes have one intron, while *ScGRAS5*, *ScGRAS33*, *ScGRAS50*, *ScGRAS56*, and *ScGRAS63* possess two introns. *ScGRAS23*, *ScGRAS43*, and *ScGRAS67* have up to four introns. Genes without introns exhibit compact structures and are widely distributed across all subfamilies, except for the Os43 subfamily, primarily in the LISCL subfamily. The DLT, LAS, DELLA, OS4, OS19, SCL4/7, SHR, and SCR subfamilies either lack introns or contain only one. PAT1 shows greater diversity in the number of exons, with five distinct exon types. Additionally, members within the same subfamily share similar gene structures, albeit with inconsistent exon and intron distributions.

The motif analysis of the 67 ScGRAS proteins using MEME online software revealed ten conserved motifs (Fig. 2C, Table S2 ~ S4). These motifs exhibited varying distribution patterns among ScGRASs, with motifs 2, 3, and 4 being widespread, except in ScGRAS2, ScGRAS15, and ScGRAS43. Motifs 10, 6, 5, and 2 were often located in close proximity across most members. Generally, ScGRAS members within the same subfamily displayed similar motif compositions. The motifs 10, 6, 5, 2, 7, 3, and 4 were present in the DLT, LAS, LISCL, OS4, OS43, SCR, and SHR subfamilies. The DELLA, DLT, LISCL, OS4, OS43, SCL3, and SCR subfamilies shared motifs 3, 1, and 4. Certain subfamilies may lack specific motif compositions, such as the absence of motifs 8 and 9 in the Os19 subfamily. Additionally, specific motifs consistently occupy particular positions within the structures of these ScGRAS proteins. Motifs 10 and 6 consistently appear at the N-terminus of proteins in subfamilies DELLA, DLT, LAS, OS4, OS43, SCL3, SCL4/7, SCR, and SHR. Motif 2 is predominantly located at the beginning of OS19. Motif 4 is usually found near the C-terminus. Overall, the motif arrangement is generally similar within members of the same subfamily, supporting the classification observed in the phylogenetic trees. We further analyzed the conservation of specific amino acids in these motifs. Overall, some conserved amino acid sites have been identified (Figure S2, Table S3).

A total of 107 cis-regulatory elements, encompassing 46 distinct physiological functions, were identified (Table S5). These elements were classified into eight categories: development-related, light-responsive, site-binding, environmental stress-responsive, promoter-related, hormone-responsive, wound-responsive, and other elements. Among the promoter elements, light-responsive elements accounted for the largest proportion, including 25 cis-regulatory factors. Promoter-related elements, such as the TATA-box, were present in all *ScGRAS* genes. Sixteen hormone-responsive elements were identified, including those responsive to abscisic acid (AAGAA-motif, ABRE related), auxin (AuxRR-core, TGA-element, AuxRE, TGA-box), gibberellin (P-box, GARE-motif, TATC-box), MeJA (TGACG-motif, CGTCA-motif), and salicylic acid (TCA-element). Moreover, several cis-regulatory elements associated with anaerobic induction, drought, fungal elicitors, anoxic-specific inducibility, low-temperature, defense responses, and stress responsiveness were also discovered. Abscisic acid-responsive elements were present in nearly 98.51% of *ScGRAS* genes, while gibberellin-responsive elements existed in 61.19% of members, and auxin-responsive elements were found in approximately 44.78%. Twelve cis-acting elements were involved in the regulatory processes of different tissues (meristem, endosperm, root, leaf, and seed) during development in *S. cereale*. Consequently, *ScGRAS* genes are implicated not only in tissue development but also in responses to various abiotic stresses. It is worth pointing out that we have found that some cis acting elements may be unique to certain subfamilies. TGA-box was found to exist only in the DELLA subfamily (*ScGRAS60* / *ScGRAS61*), which is an auxin responsive element, suggesting that the physiological functions of members of the DELLA subfamily may be complex. GATT-motif is only found in the HAM subfamily, which is a part of a light responsive element.

### Chromosomal spread and gene duplication of *ScGRAS* genes

**Fig. 3:**
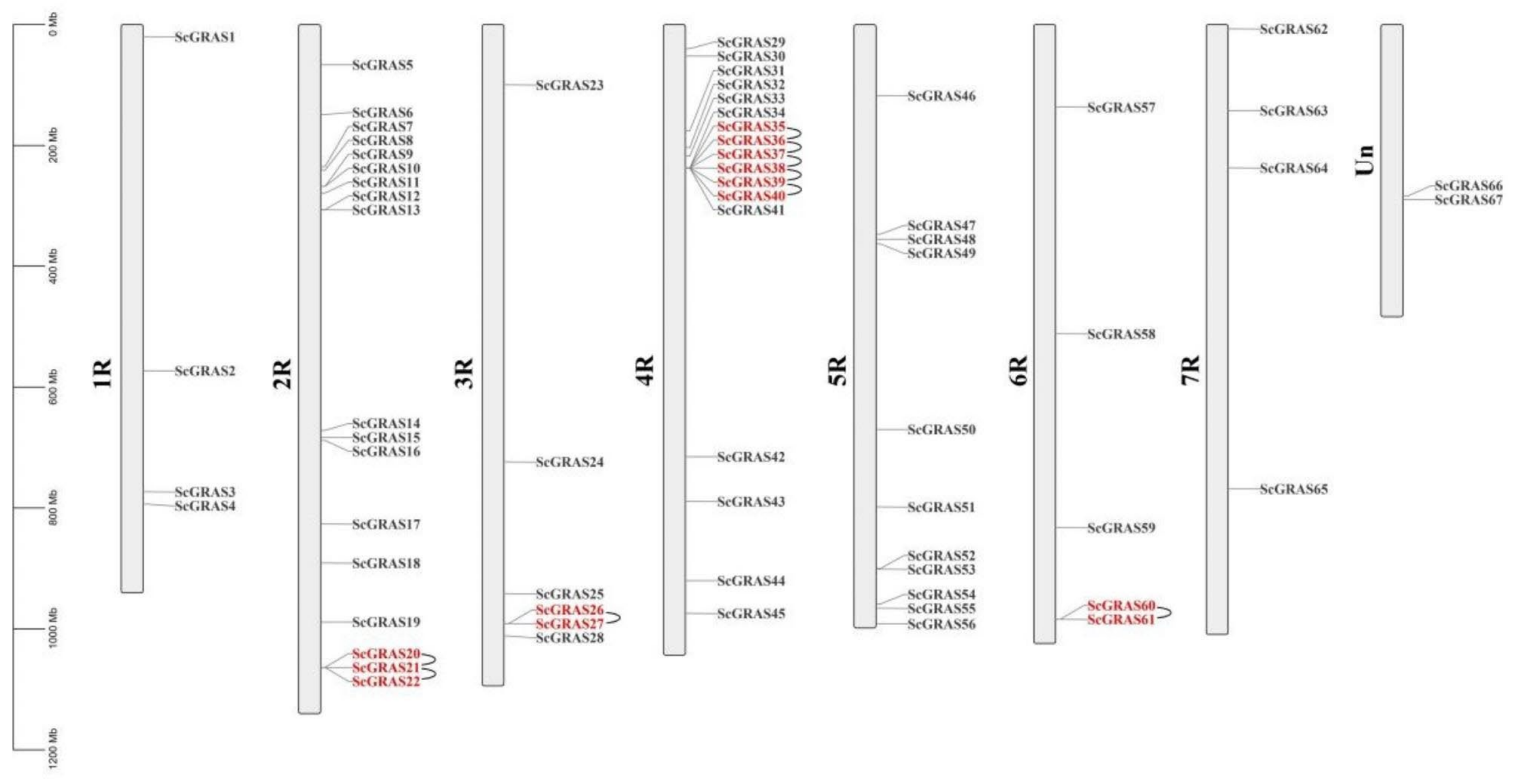
Schematic representation of the chromosomal distribution of the *S. cereale GRAS* genes. Vertical bars represent the chromosomes of *S. cereale*. The chromosome number is indicated to the left of each chromosome. The scale on the left represents chromosome length. Gene pairs with tandem repeat relationships are marked in red. The tandem gene pairs between pairs are connected by U-shaped lines

**Fig. 4:**
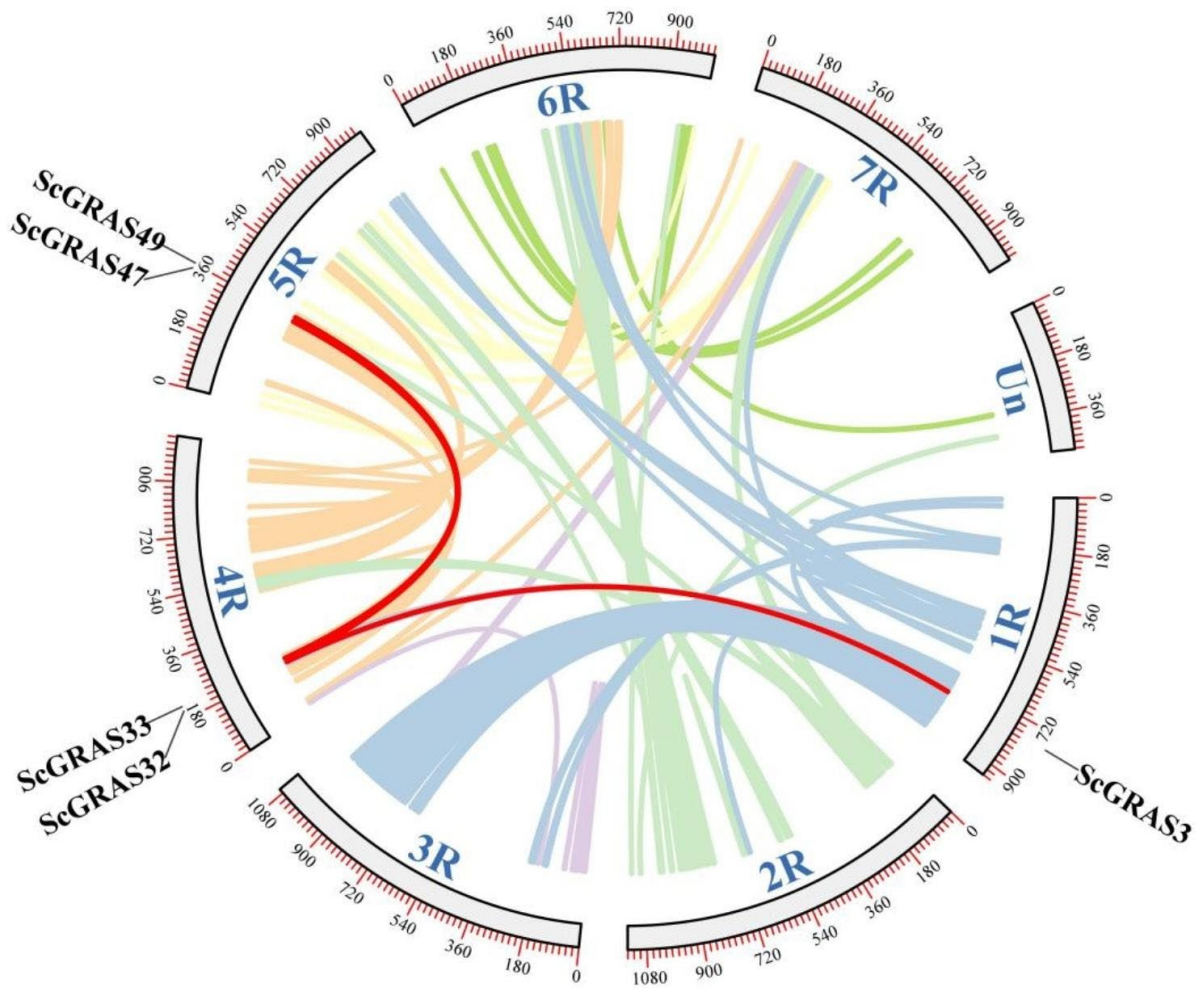
Schematic representation of the chromosomal distribution and interchromosomal relationships of *S. cereale GRAS* genes. Colored lines indicate all synteny blocks in the *S. cereale* genome, and the red lines indicate duplicated *GRAS* gene pairs. The chromosome number is indicated at the bottom of each chromosome

The 65 *ScGRAS* genes are unevenly distributed across chromosomes 1R to 7R (Fig. 3, Table S6). Additionally, two *ScGRAS* genes (*ScGRAS66* and *ScGRAS67*) were located on unassigned chromosomes (Un) Chromosome 2R contained the highest number of *ScGRAS* genes (18 genes, ~ 26.87%), followed by 4R (17 genes, ~ 25.37%). The lowest numbers were observed on 1R and 7R (4 genes, ~ 5.97%). Chromosomes 6R, 3R, and 5R harbored 5 (~ 7.46%), 6 (~ 8.96%), and 11 (~ 16.42%) *ScGRAS* genes, respectively. Nine gene duplication events were detected within the *GRAS* gene family in *S. cereale*. Tandem repeat events were observed on chromosomes 2R, 3R, 4R, and 6R, particularly in *ScGRAS36*, *ScGRAS37*, *ScGRAS38*, and *ScGRAS39*. A region enriched with tandem repeats was identified, encompassing genes *ScGRAS35* to *ScGRAS40*, all belonging to the LISCL subfamily. Three pairs of segmental duplications involving *ScGRAS* genes were detected (Fig. 4, Table S8). Five collateral homologs were identified in *ScGRAS* genes, accounting for 8.96% of the total, suggesting that these genes may have originated from segmental expansion events. In general, the typical domain of *GRAS* family is a VHIID motif flanked by two Leucine rich regions. The ‘VHIID’ motif represents several important amino acids. However, the core regions of these proteins are replaced by ‘LHIVD’. Except for ScGRAS35, the SAW motifs of other members are composed of three conserved amino acid residues: R (x4) E, W (x7) G, and W (x10) W structures. ScGRAS37/SCGRAS38 (86.2%) and ScGRAS39/SCGRAS40 (85.7%) had high similarity (Table S7). Chromosome 4R contained the most *ScGRAS* members (n = 3). In contrast to tandem duplication, two homologous expansion events involving four genes (*ScGRAS32* / *ScGRAS47*, and *ScGRAS33* / *ScGRAS49*) were discovered. These segmental duplications primarily involved the SCR and SCL3 subfamilies, while other groups exhibited greater conservation during evolution.

### Synteny analysis of *ScGRAS* genes

**Fig. 5:**
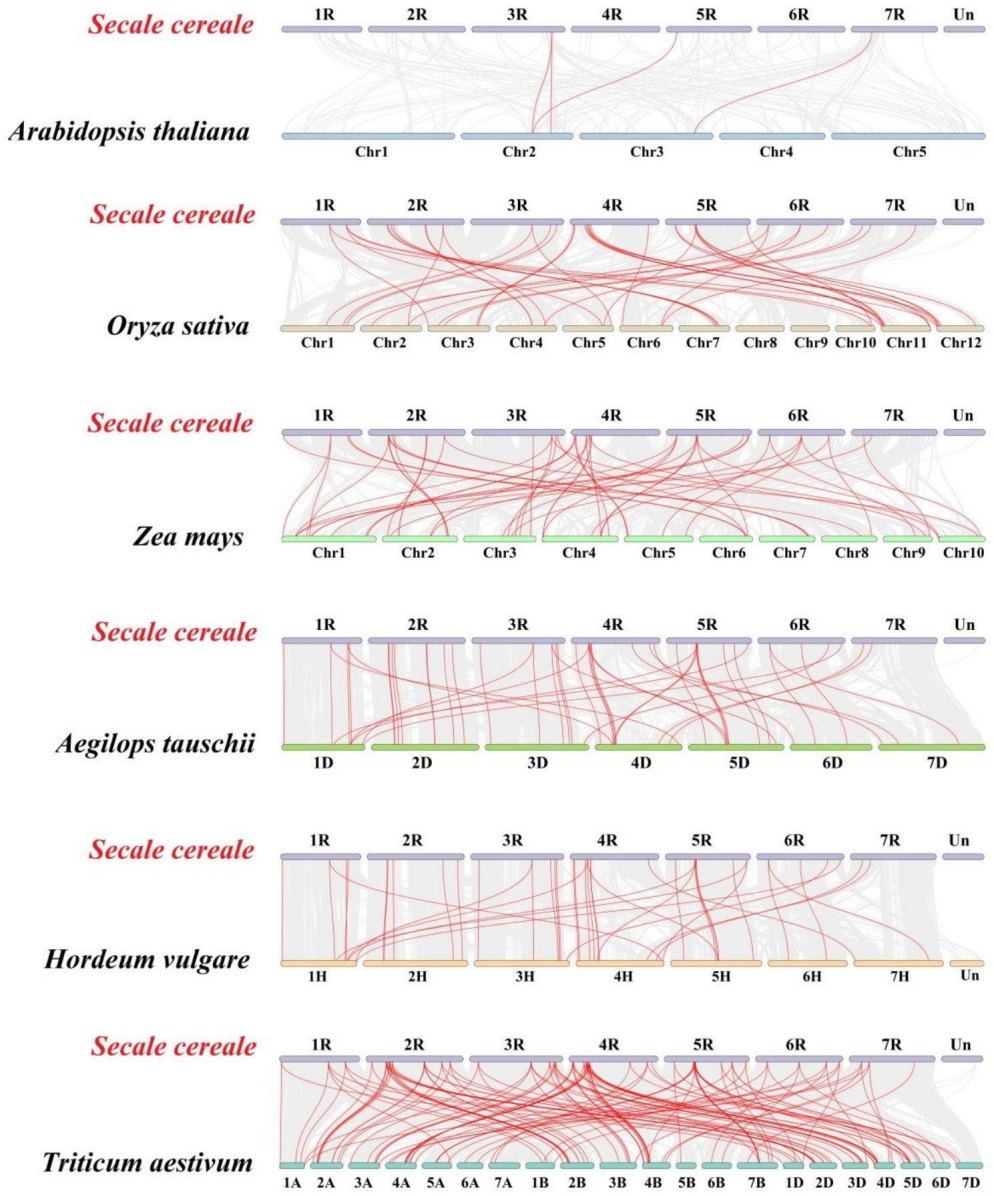
Synteny analyses of the *GRAS* genes between *Secale cereale* and six representative plant species (*Triticum aestivum*, *Aegilops tauschii*, *Hordeum vulgare*, *Oryza sativa* subsp. *Indica*, *Zea mays*, and *Arabidopsi thaliana*). Gray lines on the background indicate the collinear blocks in *S. cereale* and other plant genomes; red lines highlight the syntenic *S. cereale GRAS* gene pairs

A total of 52 *ScGRAS* genes showed homologous relationships with genes in *A. thaliana* (n = 3), *O. sativa* (n = 30), *Z. mays* (n = 34), *Aegilops tauschii* (n = 39), *H. vulgare* (n = 35), and *T. aestivum* (n = 49) (Fig. 5, Table S9). The number of collinear gene pairs between rye and other representative species (*A. thaliana*, *O. sativa*, *Z. mays*, *A. tauschii*, *T. aestivum*, and *H. vulgare*) were 4, 42, 54, 49, 42, and 137, respectively. Rye exhibited a relatively high proportion of *GRAS* gene orthologous pairs with *A. tauschii* and *H. vulgare*, accounting for 79.59% and 83.33%, respectively. Some homologous gene pairs between rye and Triticeae Dumortier plants were not identified in *A. thaliana*, *O. sativa*, and *Z. mays*. For example, *ScGRAS5* had homologs *AET1Gv20229700* / *ARI1A01G110900* / *HORVU1Hr1G020370*, indicating possible expansion events specific to Triticeae Dumortier plants that differ from dicotyledonous plants like *Arabidopsis* and other monocotyledonous plants. Moreover, collateral homologous pairs were observed among dicotyledonous and monocotyledonous plants, with genes such as *ScGRAS25*, *ScGRAS46*, and *ScGRAS64* suggesting ancestral origins before plant differentiation. Tajima-D neutrality testing was conducted on the 67 *ScGRAS* genes to better understand their targeted or balanced selection. The D value obtained was 7.49 (Table S10), significantly deviating from zero, indicating the involvement of the *ScGRAS* gene family in evolutionary neutral selection. Furthermore, we evaluated the Ka/Ks values within these subfamilies. This calculation will help estimate the selection pressure acting on these duplicated genes, advancing insights into three categories of selection: purifying, positive, and neutral. The results showed that most genes were subjected to purification selection (Table S11). This result also exists in most genes involved in repetitive events.

### Evolutionary analysis of *ScGRAS* and *GRAS* genes of several different species

**Fig. 6:**
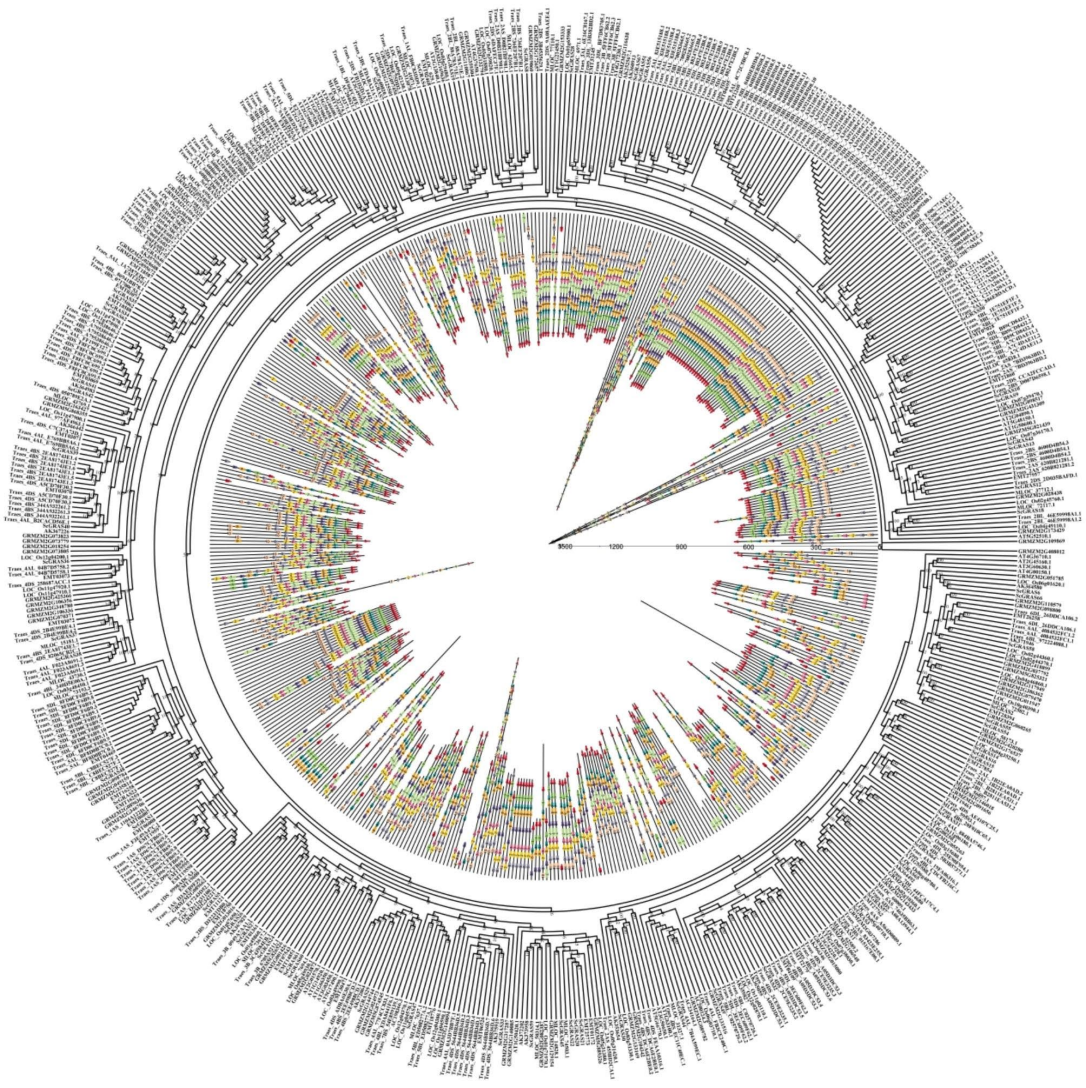
Phylogenetic relationship and motif composition of the *GRAS* proteins from *S. cereale* with six different plant species (*T. aestivum*, *A. tauschii*, *H. vulgare*, *O. sativa* subsp. *Indica*, *Z. mays*, and *A. thaliana*). Outer panel: an unrooted phylogenetic tree constructed using Geneious R11 with the neighbor-joining method. Inner panel: distribution of conserved motifs in *GRAS* proteins. The differently colored boxes represent different motifs and their positions in each *GRAS* protein sequence. The sequence information for each motif is provided in Table S2

To analyze the genetic relationship between GRAS proteins in rye and six representative plants (*A. thaliana*, *O. sativa*, *Z. mays*, *A. tauschii*, *T. aestivum*, and *H. vulgare*), an unrooted NJ tree was constructed. Ten conserved motifs were identified in the sequences of 601 GRAS proteins from these plants using MEME online service software (Fig. 6 and S3, Table S2 ~ S4). Detailed genetic correspondences are provided in Tables S1 and S2. ScGRAS proteins tend to cluster with GRAS members of *A. tauschii*, *T. aestivum*, and *H. vulgare*. With a few exceptions such as ScGRAS14, ScGRAS16, ScGRAS43, and ScGRAS67, all other ScGRAS proteins contain motifs 2 and 3. The arrangements and structures of certain motifs exhibit specificity, differentiating genes from various subfamilies and forming distinct topological patterns. Motifs 1, 8, and 9 are absent in the subfamilies HAM and LAS. Members of the subfamily OS19 (ScGRAS14, ScGRAS15, and ScGRAS16) lack motifs 1, 7, 8, and 9. Overall, GRAS genes from Triticeae Dumortier plants and *S. cereale* that occupy the same topological branches share similar motif arrangements. Specific GRAS protein subfamilies in these plants often possess analogous motifs, indicating their evolutionary relationship. Motifs 8, 4, and 5 form a conserved structure and tend to cluster within the HAM and LAS subfamilies, while motifs 3, 7, 9, 8, 4, 1, and 5 tend to aggregate within the subfamilies DELLA, DLT, LISCL, OS4, OS43, PAT1, and SCL4/7.

### Expression patterns of *ScGRAS*s in several plant organs

**Fig. 7:**
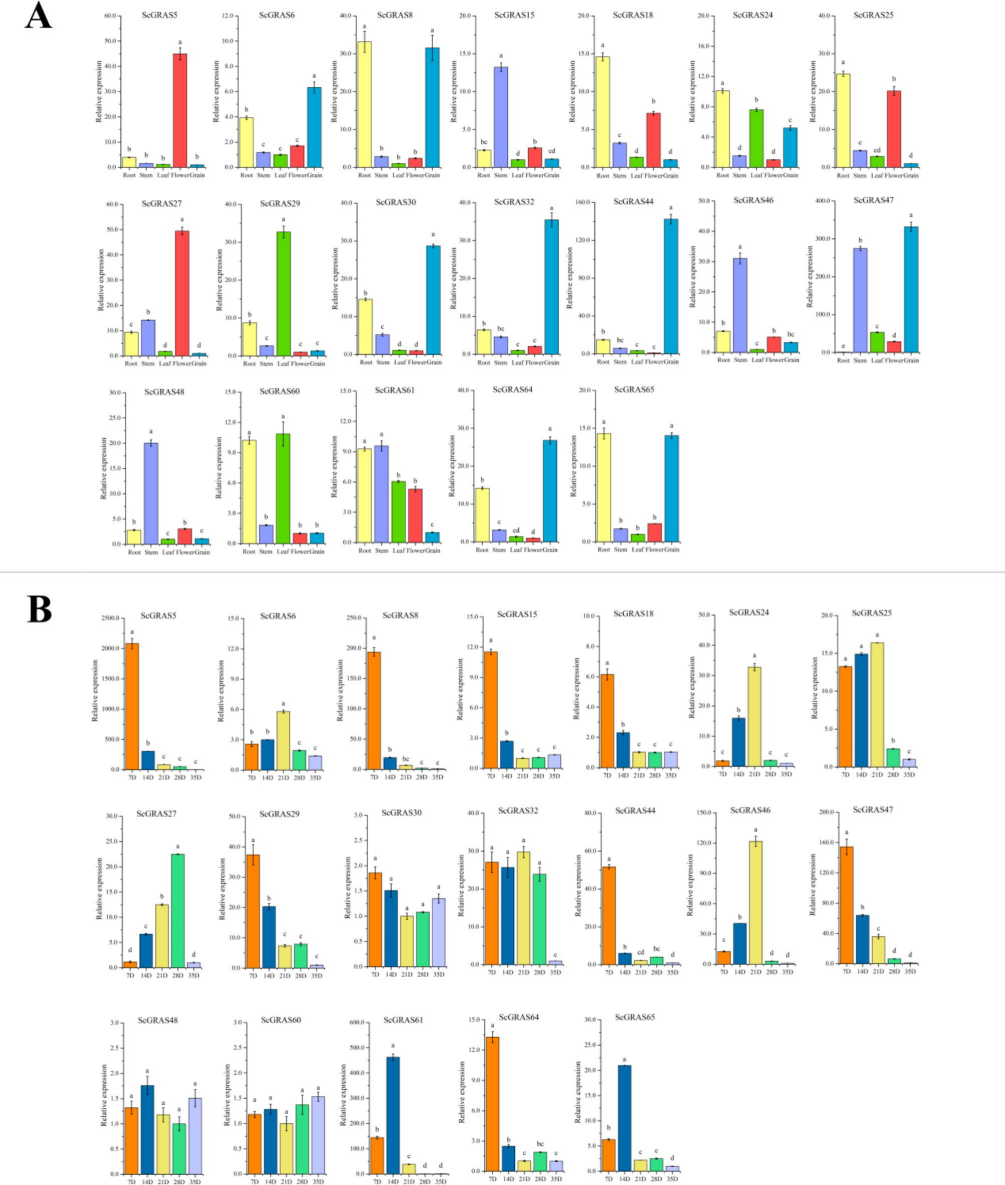
Expression patterns of selected 19 *S. cereale GRAS* genes. **A** Expression patterns of 19 *S. cereale GRAS* genes in the root, stem, leave, flower, and grain were examined via qRT-PCR. Relative expression level was shown as mean (± SE) from three independent experiments. **B** Expression patterns of 19 *S. cereale GRAS* genes were examined during different grain development stages: 7 DPA (early-filling stage), 14 DPA (mid-filling stage), 21 DPA (early-ripening stage), 28 DPA (mid-ripening stage), and 35 DPA (full-ripening stage). Lowercase letters above the bars indicate significant differences (α = 0.05, LSD) among the treatments

To investigate the physiological functions of *GRAS* genes in rye, real-time PCR was employed to detect the expression levels of 19 members during the 21 DPA (days post-anthesis) of rye grains. Transcript accumulation in five organs (leaves, stems, roots, flowers, and grains) was assessed (Fig. 7A). Most *ScGRAS* members exhibited preferential expression in specific tissues. The highest expression was observed in roots for seven genes (*ScGRAS8*, *ScGRAS18*, *ScGRAS24*, *ScGRAS25*, *ScGRAS60*, *ScGRA61*, and *ScGRAS65*), in stems for five genes (*ScGRAS15*, *ScGRAS46*, *ScGRAS47*, *ScGRAS48*, and *ScGRAS61*), in leaves for two genes (*ScGRAS29* and *ScGRAS61*), in flowers for two genes (*ScGRAS5* and *ScGRAS27*), and in grains for eight genes (*ScGRAS6*, *ScGRAS8*, *ScGRAS30*, *ScGRAS32*, *ScGRAS44*, *ScGRAS47*, *ScGRAS64*, and *ScGRAS65*).

Expression levels of most *ScGRAS* genes varied significantly at different stages of grain development. In general, gene expression was higher before the early ripening stage (21 DPA) compared to the mid-full filling stages. Nine genes (*ScGRAS5*, *ScGRAS8*, *ScGRAS15*, *ScGRAS18*, *ScGRAS25*, *ScGRAS29*, *ScGRAS44*, *ScGRAS47*) exhibited highest expression at 7 DPA, while four genes (*ScGRAS24, ScGRAS25*, *ScGRAS32*, and *ScGRAS46*) highest expression at 21 DPA (Fig. 7B). Except for *ScGRAS30*, *ScGRAS48*, and *ScGRAS60*, most genes exhibited stable expression levels in grains, with the lowest expression generally observed during the fully ripened stage (35 DPA).

Furthermore, certain *ScGRAS* members displayed coordinated expression patterns across multiple plant organs. The expression levels of some *GRAS* members exhibited significant positive correlations. For example, *ScGRAS6*, *ScGRAS8*, *ScGRAS30*, *ScGRAS32*, *ScGRAS44*, *ScGRAS64*, and *ScGRAS65* were co-expressed in various plant organs (Figure S4), while *ScGRAS5*, *ScGRAS8*, *ScGRAS15*, *ScGRAS18*, *ScGRAS29*, *ScGRAS30*, *ScGRAS44*, *ScGRAS47*, and *ScGRAS64* were co-expressed in grains (Figure S5). Notably, within the DELLA subfamily, the expression levels of *ScGRAS24*, *ScGRAS29*, and *ScGRAS60* exhibited a significant positive correlation in different tissues.

### Effects of grain developments and expression of DELLA subfamily genes after paclobutrazol and gibberellin treatments

**Fig. 8:**
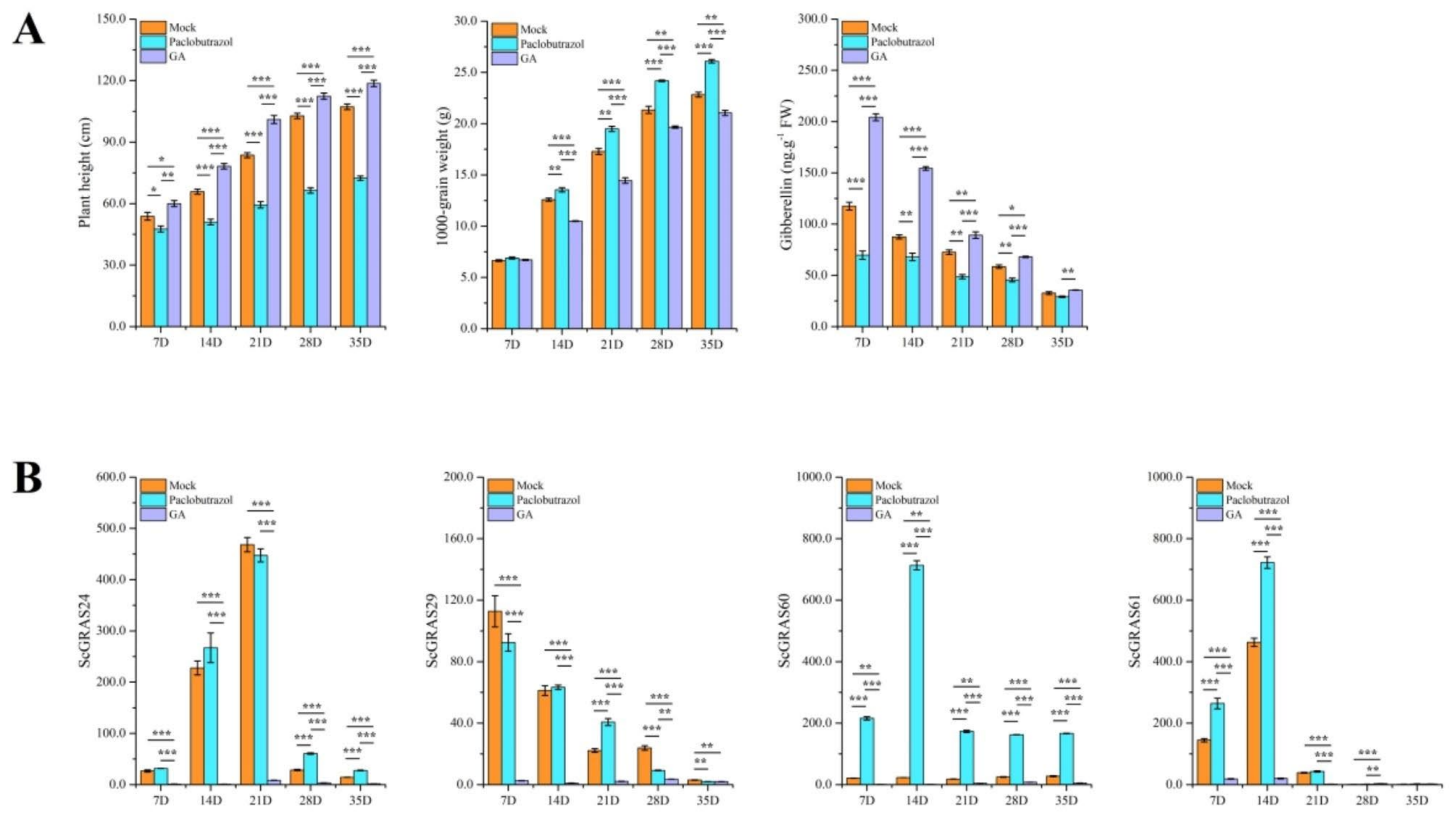
Grain development of *S. cereale* under exogenous paclobutrazol and gibberellin treatment. **A** The plant height, 1000 grain weight, and gibberellin content during grain development. **B** Differences in the expression of DELLA subfamily genes under exogenous paclobutrazol and gibberellin treatment during grain development. Mock: the same amount of water treatment, Paclobutrazol: 250 mg/L paclobutrazol treatment. Gibberellin: 100 μm gibberellin treatment. Error bars were obtained from three measurements. We need information that asterisk described significant differences (α = 0.05/0.01/0.001, LSD) among the treatments. *, **, and *** indicate significant correlations at the 0.05, 0.01 and 0.001 levels, respectively

Compared to the control (Mock), plant height in rye significantly decreased with paclobutrazol treatment, while grain filling was promoted (Fig. 8A). This effect was particularly noticeable during the later stages of grain development. As the grain-filling process advanced, endogenous gibberellin content gradually decreased in all groups, including the treatment and control groups. The gibberellin content in the paclobutrazol treatment group exhibited a rapid decline at 14 DPA and 21 DPA, stabilizing thereafter at 35 DPA. Interestingly, plant height in rye significantly increased during gibberellin treatment, particularly during the middle and late stages of grain filling, while the 1000-grain weight significantly decreased. These findings suggest that paclobutrazol primarily influences the filling process through the gibberellin pathway in rye.

Exogenous paclobutrazol and gibberellin treatments significantly influenced the expression of DELLA subfamily genes in rye (Fig. 8B). Expression levels of *ScGRAS24*, *ScGRAS60*, and *ScGRAS61* demonstrated an initial increase followed by gradual decline, reaching their lowest values at 35 DPA. *ScGRAS29*, on the other hand, exhibited a steady decrease in expression. Moreover, most DELLA members showed significant down-regulation during the filling period following gibberellin treatment, indicating a potential antagonistic relationship. The expression of *ScGRAS24* remained unchanged in the early stages of grain filling, highest expression at 28 DPA in the paclobutrazol treatment, suggesting its potential role in later filling stages. *ScGRAS61* expression significantly increased at 7 DPA, reaching its highest expression at 14 DPA. Interestingly, *ScGRAS60* expression was significantly up-regulated in nearly all induction treatments, indicating its sensitivity to paclobutrazol.

### Expression patterns of *ScGRAS* genes in response to different treatments

Numerous *ScGRAS* members exhibited significant up-regulation or inhibition under various stress conditions due to specific treatments (Figure S6). The expression of *ScGRAS6* and *ScGRAS24* significantly increased in roots, stems, and leaves after one hour of cold stress. The expression of certain *GRAS* genes displayed dynamic patterns, with differential expression levels observed across organs or treatment durations. *ScGRAS6*, *ScGRAS24*, and *ScGRAS60* were significantly up-regulated and subsequently down-regulated under heat stress. *ScGRAS5* expression gradually increased in roots while decreasing in stems and leaves. Many *ScGRAS* genes showed contrasting expression patterns under different stress treatments. The expression of *ScGRAS6*, *ScGRAS8*, and *ScGRAS24* was significantly up-regulated initially and then down-regulated in stems following UV-A, flooding, and heat treatments. Other genes exhibited distinct characteristics within specific tissues and exposure times. *ScGRAS47* displayed significant responses to cold and NaCl treatments in roots and stems but exhibited no change in leaves. The correlations between the expression patterns of *ScGRAS* genes were observed (Figure S7). Most *ScGRAS* genes exhibited negative correlations, although certain genes demonstrated significant positive correlations, such as *ScGRAS5*, *ScGRAS6*, *ScGRAS25*, and *ScGRAS47* (*P* < 0.05).

Expression patterns of *GRAS* members during different stages of grain development were analyzed under various treatments. All genes containing corresponding hormone-responsive elements in their promoter regions were detected (Figure S8, Table S12). Based on significant correlation connections used to construct a network, it becomes evident that the expression of some genes may be synergistic. Positive co-expression was observed among *ScGRAS8*, *ScGRAS15*, *ScGRAS18*, *ScGRAS27*, *ScGRAS46*, *ScGRAS64*, and *ScGRAS65* under abscisic acid induction. Similarly, *ScGRAS6*, *ScGRAS27*, *ScGRAS30*, *ScGRAS32*, *ScGRAS61*, and *ScGRAS64* showed positive co-expression under auxin induction. The expression patterns of DELLA family members did not consistently align with gibberellin and paclobutrazol induction, suggesting diverse functions. Although some co-expressed genes might interact, such as *ScGRAS65* exhibiting positive correlation with *ScGRAS24*, *ScGRAS29*, and *ScGRAS61* under gibberellin induction, these results underscore the complexity of physiological functions within different subfamilies of the *GRAS* family.

## Discussion

### *ScGRAS* gene structures and evolutionary analyses

The GRAS proteins in rye exhibit considerable structural diversity, particularly among the thirteen subfamilies, indicating that the physiological function of the GRAS gene family in rye is complex (Fig. 1 and S1, Table S1). The proportion of GRAS genes in the rye genome is approximately 0.15%, which is lower than that in other plants such as *G. max* (0.21%) [27], *V. vinifera* (0.17%) [41], *H. vulgare* (0.16%) [28], *S. italica* (0.16%) [37], *S. bicolor* (0.24%) [39], *T. aestivum* (0.17%) [40], *Z. mays* (0.22%) [42], but higher than that in *A. thaliana* (0.11%) [19]. Within the *GRAS* gene family of rye, there are thirteen subfamilies, including DELLA, DLT, HAM, LISCL, LAS, SCL3, SCL4/7, SCR, SHR, PAT1, OS4, OS43, and OS19 (Fig. 1, Table S1). It is speculated that these thirteen subfamilies may be present in most Gramineae plants and have fundamental physiological functions that are conserved throughout evolution [34]. Furthermore, the classification of the GRAS gene family may have become fixed in early higher plants and remained unchanged during plant evolution. However, the ancestral proteins within this family may continue to evolve, resulting in expansion and the emergence of new physiological functions in subsequent plant generations, depending on the specific plant species and environmental conditions [34]. These certain ScGRAS proteins (ScGRAS5, ScGRAS14, ScGRAS15, ScGRAS16, ScGRAS26, and ScGRAS27) have been classified into rice-specific subfamilies, indicating that the GRAS family may undergo further differentiation in monocotyledonous plants. Among the subfamilies, LISCL have the highest number of members (18, ~ 26.87%), while OS43 (ScGRAS5), SCL4/7 (ScGRAS30), and DLT (ScGRAS44) have the fewest members. Similarly to other plants such as *Arabidopsis* [19], rice [34], *S. italica* [37], *S. bicolor* [39], *T. aestivum* [40], and *Z. mays* [42], many subfamilies within the GRAS gene family of rye are likely to be conserved, whereas LISCL may exhibit greater differentiation ability. The differences in expansion among these subfamilies are speculated to be associated with the physiological functions of different proteins and their adaptation to the environment during evolution. However, more research is needed to determine whether the structural differences among these subfamilies are related to environmental adaptation. To further analyze the *GRAS* gene family in rye from different sources, we identified another important rye genome (Lo7) [67]. A total of 72 independent GRAS proteins were identified in the ‘Lo7’. Similarly, these genes were primarily classified into 13 typical subfamilies (Figure S9, Table S13). The GRAS proteins of ‘Weining’ was used to co construct the evolutionary tree, which was consistent with our original classification. To explain the differences and homology among these members, we constructed a comparative genome in the two rye (Figure S10, Table S14). Most genes were assigned to the corresponding chromosomes (1R ~ 7R), indicating the overall reliability of the results. However, we observed that there are still some genes that have not been defined as corresponding homologues. We speculate that this may be a difference in genome assembly.

Most of these GRAS genes in rye contain conserved domains, including LHR I, VHIID, LHR II, PFYRE, and SAW. As shown in Figure S1, the VHIID domain is considered the central region and contains highly conserved histidine and aspartic acid residues, which serve as the base and supporting sites of GRAS proteins [76–78]. There may be cross-substitution of non-polar hydrophobic amino acid residues, such as histidine (His), leucine (Leu), isoleucine (Ile), and valine (Val), within the core region. These substitutions are likely the result of genetic mutations, although they may not significantly alter the peptide chain structure [79]. Furthermore, some GRAS proteins belonging to the LISCL subfamily (ScGRAS34, ScGRAS35, and ScGRAS62) and PAT1 subfamily (ScGRAS43) do not contain conserved histidine and aspartic acid residues in the VHIID region. The structural differences of these genes may indicate further differentiation of GRAS proteins, as also observed in sorghum [39]. There are numerous variations in amino acid residues within the VHIID region of the LISCL and PAT1 subfamilies. It is speculated that the high activity of the LISCL and PAT1 subfamilies leads to structural differentiation in the domains, resulting in amino acid instability. This phenomenon may explain why these subfamilies have expanded and become the largest subfamily. Some conserved amino acid segments in the structural domain of ScGRAS43, the member of the PAT1 subfamily, have been lost, possibly due to chromosome fragment translocation or inversion [11, 80]. The acquisition and loss of structural domains are important driving forces for gene family expansion, as observed in other higher plants such as sorghum [37] and maize [42]. Inherently disordered regions, which can undergo conformational changes between order and disorder, are abundant in eukaryotic proteomes [37, 39]. These functional regions, which contain short molecular recognition features (MORFs) in the N-terminal structural domain of GRAS proteins, play crucial roles in cell signal transduction and transcriptional regulation. Therefore, GRAS proteins possess functional specificity [16]. Although the N-terminus of GRAS proteins exhibits high variability, some residues display similarities across different subfamilies. For example, the DELLA subfamily protein contains the DELL A structural domain at its N-terminus.

The introns of these *ScGRAS* genes were examined, and it was found that each gene contains between 1 and 5 exons (Fig. 2A and B). Approximately 59.7% of *ScGRAS* genes do not contain introns, which is higher than in rice (~ 55%) [34] and poplar (~ 54.7%) [65], but lower than in millet (~ 64.9%) [37], sorghum (~ 66.7%) [39], *Arabidopsis* (~ 67.6%) [19], and buckwheat (~ 87%) [26]. The gene structure of certain subfamily members may be compact, as some subfamilies such as DLT, LAS, and DELLA do not contain introns or have only one intron. Genes without introns are also observed in other gene families, including the small auxin-up RNA (SAUR) gene family [81], F-box families [82], and DEAD box RNA helicase [83]. Generally, genes without introns or with few introns tend to have lower expression levels in plants. However, it has been suggested that *GRAS* genes in plants may have originated directly from prokaryotes through horizontal gene transfer and duplication events [42]. Therefore, most GRAS members in plants may have compact gene structures [84]. Genes without introns can continuously encode proteins during transcription and translation, making them sensitive to the environment and capable of responding rapidly [85–87]. Furthermore, gene expression may not strongly depend on the density of introns in these genes, as evidenced by our research results [68]. Some highly expressed genes have introns of average length (Fig. 7 and S4), indicating that the expression level may depend on specific developmental processes or environmental stress [69]. For example, the expression of *ScGRAS64* in leaves increases rapidly under cold, salt, and PEG stresses, suggesting that it may be a response to these abiotic stresses. Genes with compact structures may contribute to rapid responses to stress or tissue development. Ten conserved motifs were identified in ScGRAS proteins, which can be used to predict the function of unknown proteins within the same subfamily [39].

Tandemly repeated genes can rapidly expand or contract in response to environmental changes, maintaining a constant number of functionally related genes without increasing genetic complexity during evolution [88]. Segmental duplications are also common in animal and plant genomes, contributing to genetic diversity [37]. Thus, tandem repeats and segmental duplications play important roles in the expansion of gene families and genome evolution, enabling plants to adapt to their environment. For example, duplication events of *OsSHR1* led to diversification, and the expression of *OsSHR2* expanded in the endodermis and certain cortex cell layers, possibly acquiring additional functions in rice root development [89]. In our study, nine tandem repeat events involving thirteen *ScGRAS* genes were identified (Fig. 3, Table S6). Notably, a region of high-density tandem repeats was found on chromosome 4R, involving four members (*ScGRAS36*, *ScGRAS37*, *ScGRAS38*, and *ScGRAS39*) belonging to the LISCL subfamily. This may explain why LISCL is the largest subfamily in the *ScGRAS* family. Furthermore, three pairs of segmental duplications were observed in *ScGRAS* genes (Fig. 4, Table S8). Consistent with other plants such as *Arabidopsis* [19], rice [34], millet [37], and barley [62], all duplicated genes are within the same subfamily, indicating that duplication events do not occur between different subfamilies. However, tandem replication of *ScGRAS* genes may be a more significant contributor to the expansion of the *GRAS* gene family in *S. cereale*, which is distinct from *S. italica* [37] and may represent a unique evolutionary pattern in rye.

### Expression patterns and function prediction of *ScGRAS* genes

The gene expression patterns were analyzed to preliminarily predict the physiological functions of these *GRAS* genes in rye. The expression of nineteen *GRAS* members was analyzed in different tissues and at different stages of grain filling (Fig. 7). Almost all *ScGRAS* genes exhibited significant differential expression (*p* < 0.05). *ScGRAS25*, encoding a member of the LISCL, displayed specific expression in roots and flowers, consistent with the homologous gene *At2G29060* in *Arabidopsis*, which participates in root, flower, and seed development. Notably, *ScGRAS18*, encoding a member of the PAT1 subfamily, is specifically expressed in roots and flowers. PAT1 members primarily participate in the signal transduction of photoreceptor A, as demonstrated by the elongation of hypocotyls, closure of apical hooks, and folded cotyledons observed in the *pat1* mutant under far-red light conditions in *Arabidopsis* [90]. *ScGRAS46* and *ScGRAS48*, both members of the same subfamily, exhibited similar expression patterns, with high expression levels in stems. Few studies have been conducted on LISCL subfamily members in higher plants, but evidence suggests that they may play roles in transcriptional regulation. The LiSCL transcription factor plays a crucial role in meiosis during the meiotic process of *L. longiflorum* [55]. Similarly, *PrSCL1* in *Pinus radiata* and *CsSCL1* in *Castanea sativa* are mainly expressed in stems and roots, induced by exogenous auxin during cutting, and involved in early adventitious root formation [91]. Furthermore, the expression pattern of *ScGRA44* was similar to that of *GS6*, a homologous gene belonging to the DLT in rice [73]. *OsGS6*, an important domestication gene, has been found to play a significant role in reducing the size of rice grains [92]. The expression patterns of DELLA family members may be complex. For example, *GRAS24* is specifically expressed in roots, leaves, and grains, while *GRAS29* exhibits high expression levels in leaves. Therefore, it is necessary to systematically analyze their expression characteristics in different tissues and at different stages of grain development. The expression patterns of many *ScGRAS* genes showed positive correlations, indicating potential synergistic effects in five plant organs (Figure S4). These findings provide insights into the function of the *GRAS* gene family in different tissues of rye, although further experiments are needed to verify their specific functions. Grain ripening is a critical process in rye, as it adapts to unfavorable climate and soil conditions and thrives in high-altitude, mountainous, and cold regions. The entire grain ripening process was divided into five representative stages, and the expression patterns of *ScGRAS* genes were analyzed to identify key candidate genes related to grain development. Most *GRAS* genes in rye are highly expressed before the early ripening stage (21 DPA), suggesting widespread involvement of the *GRAS* family in grain ripening. For instance, *ScGRAS48* and *ScGRAS60* are stably expressed in almost all stages.

DELLA proteins not only regulate the gibberellin response pathway but also function as central hubs in signaling pathways that integrate signals from various hormones, such as jasmonic acid, auxins, abscisic acid, and ethylene [50, 51]. Gibberellins are central regulators of plant growth and behavior, acting by degrading DELLA proteins. Peng, et al. [93] proposed that *Rht-B1* / *Rht-D1* and maize *dwarf-8* are homologous to the gibberellin-insensitive (GAI) gene in *Arabidopsis*. DELLA proteins act as negative regulators in the gibberellin signaling pathway, inhibiting gene expression and plant growth. However, DELLA proteins can also be degraded by gibberellins, thereby eliminating their inhibitory effects. The degradation of DELLA proteins occurs through binding with the GA-GAI complex protein, leading to degradation and relieving inhibition by gibberellin. This balance between gibberellins and DELLA proteins forms a dynamic regulatory mechanism for gibberellin activity levels in plant growth and development [94, 95]. Furthermore, DELLA plays a crucial role in grain development. For example, the expression of *DELLA* genes in tomato and *Arabidopsis* induces parthenocarpy [96]. In rye grains, gibberellin can be detected throughout the entire developmental stage (Fig. 8). Therefore, it is hypothesized that young rye grains immediately produce gibberellin after fertilization to promote grain filling [39]. The endogenous gibberellin content in rye grains was analyzed and detected throughout the entire grain development stages, gradually decreasing as the grain ripens. In addition to *ScGRAS60*, *ScDELLAs* exhibit significantly higher expression levels during the early ripening stage (before 21 DPA) of grain development compared to the full ripening stage (35 DPA). These findings suggest that DELLA genes may play a role in the early to mid-stages of grain development. The plant growth regulator paclobutrazol, which regulates DELLA transcription and gibberellin biosynthesis, was used to treat rye plants [97]. Paclobutrazol treatment significantly reduced plant height and gibberellin content while increasing grain weight (Fig. 8B). It is speculated that under paclobutrazol treatment, more photosynthetic products are directed towards grain development rather than stem elongation [98]. Furthermore, exogenous gibberellin treatment had opposite effects on plant height and grain weight compared to paclobutrazol treatment. Paclobutrazol significantly inhibits gibberellin biosynthesis, especially during the early to middle filling stage (7 DPA and 14 DPA), potentially influencing the expression patterns of DELLA members due to the down-regulation of gibberellin. Almost all DELLAs exhibited suppressed expression levels under gibberellin treatment compared to the control group. After paclobutrazol treatment, the expression level of *ScGRAS24* changed significantly at 28 DPA and 35 DPA, indicating potential sensitivity during the full ripening stage. Conversely, the expression level of *ScGRAS61* significantly increased at 7 DPA and 14 DPA. *ScGRAS29* displayed a unique expression pattern with fluctuating levels, possibly due to significant differences in amino acid structure and motif arrangement compared to other members. Notably, the expression level of *ScGRAS60* significantly increased throughout grain development after paclobutrazol treatment and was more sensitive than other DELLA members. This suggests that *ScGRAS60* may have potential value in breeding rye. Additionally, significant differences in the expression levels of four DELLA subfamily genes were observed during grain development after paclobutrazol treatment, indicating potential functional differentiation among different members of the DELLA. This is consistent with previous findings in *Arabidopsis*, where members of the DELLA exhibit differentiated functions while retaining some overlapping functions [99].

Rye possesses the ability to adapt to unfavorable climate and soil conditions, allowing it to thrive in high-altitude areas, mountainous regions, and cold environments. This adaptation may be regulated by a complex endogenous network and transcriptional signals that enable rye to respond to abiotic stresses [100]. However, the stress response of rye to complex abiotic stresses has not been systematically analyzed. To explore the physiological roles of *GRAS* in environmental adaptation in rye, the expression of nineteen *GRAS* members in response to six different abiotic stresses and three representative hormone treatments was analyzed in rye seedlings (Figures S6 and S8). Under cold stress, the expression levels of 11 *ScGRAS* genes in roots, 12 genes in leaves, and 13 genes in stems were significantly regulated, depending on the duration of the treatment. These responses may contribute to the adaptation of *S. cereale* to cold environments, which is consistent with its role as a cold-tolerant crop, as demonstrated in grape [101] and millet [37]. The member *ScGRAS8* of the SHR subfamily showed rapid induction under UV-A treatment. In wheat, *TaSCL14* is highly expressed in stems and roots in response to high light stress. Silencing *TaSCL14* leads to decreased tolerance of wheat to high light stress, resulting in dark-induced leaf senescence and poor development [102]. *SHOOT GRAVITROPISM 1* (*At3G54220*) and *ScGRAS6* belong to the HAM and share similar basic sequence compositions. *SGR1* promotes cell elongation and endodermis differentiation outside the meristematic tissue, which is crucial for root growth. Furthermore, the *sgr1* mutant participates in the abscisic acid pathway and coordinates the oxidative stress response in plants by mediating the inhibition of cytokinin response in the meristematic tissue to promote root growth [103]. *ScGRAS18* exhibits significant induction in roots under six different abiotic stresses. In rice, *CIGR1* and *CIGR2* are rapidly induced upon perception of N-acetylchitooligosaccharides elicitor, induced by exogenous gibberellins, which may play a key role(s) as transcriptional regulators in the early stages of defense signaling following fungal perception and pathogenesis [104]. *GmGRAS37*, which responds to drought, salt stress, abscisic acid, and brassinosteroids, enhances drought and salt stress resistance when overexpressed in soybean hairy roots [105]. In tomato, the HAM member *SlGRAS40* is induced by D-mannitol and NaCl, playing a role in promoter- and auxin- and gibberellin-mediated signal transduction in response to abiotic stresses [106]. From the cluster tree, it can be seen that the HAM subfamily may have two highly differentiated sub-classes, similar to the HAM subfamily in sorghum we previously reported [39]. Therefore, two members of the HAM subfamily, *ScGRAS6* and *ScGRAS14*, were selected for expression pattern analysis. Their expression patterns differed somewhat, with *ScGRAS6* being highly expressed at 21 DPA and *ScGRAS14* being highly expressed at 7 DPA. Under heat stress, the expression of *ScGRAS6* increased and then decreased in stems, while the expression pattern of *ScGRAS14* was opposite. These findings suggest that different branches of the *GRAS* subfamily may exhibit distinct responses to environmental stress. Additionally, some genes may participate in the response to abiotic stress through hormone regulation. For example, the *AtDLT* gene regulates brassinosteroid signaling by binding in the promoter of the BZR1gene, thereby regulating leaf curling and embryo sheath elongation [107]. Overall, different *GRAS* subfamilies have diverse biological functions, playing important roles in plant adaptation to abiotic stresses. This is supported by the correlation network (Table S12, Figure S8), which demonstrates that these *GRAS* transcription factors participate in a complex cross-regulatory network induced by stress and hormones.

## Conclusion

In conclusion, this study identified 67 members of the *GRAS* gene family in rye and classified them into thirteen main subfamilies. Most *ScGRAS* genes do not contain introns, and their gene structures, conserved motifs, cis-acting elements, gene duplications, and expression patterns were analyzed. Overall, the gene structures of the same subfamily is always similar, including the number of exons, amino acid structures, and motif arrangements. Gene duplication events may have contributed to the emergence of certain *ScGRAS* genes, with tandem replication playing a more significant role in expanding the *GRAS* gene family compared to segmental duplication. Notably, a high-density tandem repeat region containing LISCL subfamily genes was discovered on chromosome 4R. The expression patterns of *ScGRAS* genes in different tissues and grain development stages were analyzed, and key candidate genes related to grain development were identified. Additionally, the relationship between DELLA genes, gibberellin content, and grain development was investigated. Furthermore, the expression patterns of *ScGRAS* genes under various abiotic stresses and hormone treatments were examined to shed light on their physiological functions in environmental adaptation. These findings provide valuable insights into the function and evolution of the *GRAS* gene family in rye.

## Methods

### Gene identification

The reference genome of rye was downloaded from the GenBank website of the National Center for Biotechnology Information, the accessed number was JADQCU000000000 [108]. Firstly, all of GRAS proteins of *Arabidopsis* and rice were used to search for candidate GRAS proteins from the rye genome via the blastp program [109]. Candidate genes were searched by blastp using a score value of ≥ 100 and e-value ≤ e − 10. Secondly, the Hidden Markov Model (HMM) file of the GRAS domain (PF03514) is downloaded from the Pfam protein family database (http://pfam.sanger.ac.uk/). Based on the HMM model in the HMMER 3.0 online software, the GRAS protein sequence in *S. cereale* was identified with a decision value of 0.01 (http://plants.ensembl.org/hmmer/index.html) [110]. Based on PFAM and SMART in thread sequencing, conserved motifs were found in the GRAS proteins in rye (http://smart.embl-heidelberg.de/) [111, 112]. Then, in the NCBI protein database, these ScGRAS proteins were used as the initial query for re-verification (https://blast.ncbi.nlm.nih.gov/Blast.cgi? PROGRAM = blastp&PAGE_TYPE = BlastSearch&LINK_LOC = blasthome). Finally, the ExPasy online program was used to identify the basic features of the *GRAS* genes in *S. cereale*, including sequence length, protein molecular weight, isoelectric points, and subcellular localization (http://web.expasy.org/protparam/). In addition, to further compare the similarity of these genes, we conducted pairwise sequence alignments on these proteins using the EMBOSS Need online website (https://www.ebi.ac.uk/Tools/psa/emboss_needle/).

### *GRAS* gene structures and conserved motif analysis

Firstly, we conducted multiple sequence alignment projects on these GRAS proteins from rye to further analyze LHR I, VHIID, LHR II, PFYRE, and SAW domains within conserved domain intervals [113]. MEGA 7.0 and GeneDoc 2.7 software were used to manually adjust the conserved domain segments in the amino acid sequences of these GRAS proteins to elucidate their diversity and variability [39]. Specifically, these software tools were used for sequence alignment and manual modification of amino acid sequences to accurately identify conserved domains [114]. Gene Structure Display Server online software was used to analyze the exon-intron substructures of these *ScGRAS* genes [37]. Additionally, the MEME online program (http://meme.nbcr.net/) was used to analyze the conserved motifs and gene structure variations among these GRAS proteins [115]. The optimized parameters for the conserved motifs were as follows: a maximum of 10 motifs and the optimal width of residues ranging from 6 to 200 [37]. Visualize Gene Structure is completed using TBtools software (v1.120) [116]. Additionally, the PlantCARE online software was used to predict the physiological functions of cis-elements in the promoter regions (up-stream 2000 bp) of these 67 *GRAS* genes [39].

### Chromosomal distribution and gene duplication

Firstly, based on the physical location of these genes in the annotation file, all *ScGRAS* genes have been designated as chromosomal details. Circos software was used to analyze these *ScGRAS* genes for chromosomal location information [117]. The presence of two or more gene members from the same family within the 200 kb chromosome region is defined as the presence of tandem repeats [37]. Multiple Collinear Scanning Toolkits (MCScanX) of TBtools software (v1.120) [116] was used with default parameters to analyze gene duplication events for these *GRAS* genes [110]. Homology of the *GRAS* genes between *S. cereale* and six other plants (*T. aestivum*, *A. tauschii*, *H. vulgare*, *O. sativa* ssp. *Indica*, *Z. mays*, and *A. thaliana*) was analyzed by using the project of dual synteny plotter in TBtools software. To further analyze the possible selection pressure in the GRAS genes of rye [118–120], the Ka/Ks values of all gene pairs in different subfamilies were calculated using the Simple Ka/Ks Calculator (NG) program of TBtools.

### Phylogenetic analysis and classification of the *ScGRAS* family

According to the classification of AtGRAS and OsGRAS proteins, 67 GRAS proteins in *S. cereale* are divided into 13 main subfamilies. In MEGA 7.0, the Jukes-Cantor model is used to construct NJ (neighbor-joining method) trees. Bootstrap value of the constructed phylogenetic tree was set to 1000, and assigned with Geneious R11 with BLOSUM62 cost matrix. To elucidate the evolutionary relationships between these GRAS proteins in several plants, the synteny maps based on homologous genes from rye and six representative plants were constructed. Five monocotyledonous plants were selected, containing three Triticeae Dumortier plants (*T. aestivum*, *A. tauschii*, *H. vulgare*), one model plant (*O. sativa*), and one C4 plant (*Z. mays*). Meanwhile, the dicotyledonous model plant (*A. thaliana*) was also included in the comparison, which was obtained from the UniProt website [32, 33].

### Plant materials, growth conditions, and abiotic stress in *S. Cereale*

*S. cereale* cv. *Weining*, a representative cultivated variety in Guizhou Province in southwest China was used. The cultivar was planted in a greenhouse at Chengdu University farm. At the early-ripening stage of rye, representative tissues were collected, including roots, stems, leaves, flowers, and grains. Additionally, to observe the expression levels of these representative genes in rye grain during the filling period, samples from five grain developmental stages were collected, i.e., 7 days (early-filling stage), 14 days (mid-filling stage), 21 days (early-ripening stage), 28 days (mid-ripening stage), and 35 days (full ripening stage). Many *ScGRAS* genes may be involved in the development of rye grains, thereby affecting the filling and nutritional structure of the grains. To determine these genes that may regulate the development of rye grains, the expression of these 19 *ScGRAS* members was evaluated during the five grain-filling stages after flowering. As far as possible, the selected members of different subfamilies exhibit significant differences in amino acid structures and distant clustering relationships. Except for the DELLA members, at least one member of different subfamilies was selected, depending on their topology and genetic structures. All the plants were grown under the same growth conditions, and these samples were collected from five plants. The collected samples were rapidly placed in liquid nitrogen and pre-cooled completely to fix their physiological status and stored at -80 °C until further use. Each sampling and stress treatment had three biological replicates. Meanwhile, these samples were performed by qRT-PCR with at least three technical repeats.

The plant RNA extraction kit (RNA Easy Fast Plant Tissue RNA Rapid Extraction Kit, DP452) was selected for total RNA extraction. In addition, to investigate the expression patterns of these *ScGRAS* genes under different abiotic stresses and hormones, seedlings of rye were subjected to abiotic stress treatment at the seedling stage (4 weeks after germination). All seedlings were planted in seedling trays, and each tray was added with 50 mL of solution to fully soak the roots of the plants. The treatment for six different abiotic stresses were UV-A radiation (70 μW/cm^2^, 67 V, 30 W), flooding (all plants), salt (5% NaCl), drought (10% PEG6000), high temperature (40℃), and low temperature (4℃). Each stress treatment was repeated three times, and samples of leaves, roots, and stems were taken at 0, 1, 4, and 12 hours for qRT-PCR analysis. Finally, considering that there were different hormone response elements in the promoter region of these genes, we conducted three different hormone treatments at the flowering stage: gibberellic acid (GA_3_, 100μM), auxin (indole-3-acetic acid, IAA, 100μM), and abscisic acid (ABA, 50μM). Paclobutrazol, a plant growth regulator, participates in the expression of members of the *GRAS* gene family [37, 39]. Therefore, it has also been considered as a candidate hormone. Whether there is a coordinated expression of these genes was observed. In addition, as a plant growth inhibitor, paclobutrazol regulates plant growth mainly by inhibiting biosynthesis of GAs by regulating DELLAs transcription [26]. In order to further investigate the relationship between DELLAs, GAs and grain development in rye, the materials of ‘Weining’ with similar growth status were selected and sprayed with 50 mL paclobutrazol (250 mg·L^− 1^) and gibberellin (100 μM) during the flowering period. Controls (mock) were sprayed with the same amount of water. Further analysis was conducted on the plant height, 1000-grain weight, gibberellin content, and gene expression level of the DELLA subfamily in control, paclobutrazol, and gibberellin-treated plants at 7, 14, 21, 28, and 35 DPA (days post-anthesis).

### Endogenous GA analysis

Regarding the method of Fan et al. [37], the gibberellic acid (GA) content in rye grains was determined. Approximately 1 g of fresh tissue from the grain was collected and ground in liquid nitrogen. The ground powder was mixed with 50 mL of 80% ethanol and subjected to ultrasonic extraction three times for 1 h each time. Supernatant was concentrated at a low temperature, then mixed with water, and N-butanol was added to extract for 1 h. Finally, the n-butanol layer was dried under a stream of nitrogen (N_2_). Ten milligrams of the dried sample were accurately weighed and dissolved in 5 mL of methanol. The dissolved solution was filtered using a 0.22 μm microporous membrane, and LC / MS was used for content detection.

### Total RNA extraction, cDNA reverse transcription, and qRT-PCR analysis

Fresh tissues of rye were extracted using a plant RNA extraction kit (RNA Easy Fast Plant Tissue RNA Rapid Extraction Kit, DP452) for total RNA extraction. Based on the primer sequences designed in Primer 5.0 software, the expression levels of different *GRAS* genes were detected (Table S15). *ACTIN* as an internal reference gene [121]. SYBR Premix ExTaqII (TaKaRa Bio) was used for standard expression detection, and experiments were performed with three replicates on a CFX96 real-time system (Bio-Rad). Real-time qPCR reaction included 40 cycles with parameter settings as follows: pre-denaturation at 95 ℃ for 30 s, denaturation at 95 ℃ for 5 s, annealing at 60 ℃ for 20 s, and extension at 72 ℃ for 20 s. All quantitative primers for genes were analyzed for their practicality through melting curves. The expression of these *GRAS* genes was analyzed using the 2^− (ΔΔCt)^ method [122].

### Statistical analysis

The least significant difference test (LSD) is further conducted using 0.05 and 0.01 significance levels to compare the means between the groups in JMP6.0 software (SAS Institute). Origin 2016 software (OriginLab Corporation, Northampton, Massachusetts, USA) has been employed to draw the histograms. Additionally, the Pearson correlation program was used to define the correlation coefficient of *ScGRAS* genes, and Sigmaplot 12.0 software (Systat Software, Inc, Point Richmond, CA) is utilized to calculate the correlation coefficient. A Pearson correlation matrix of the *GRAS* genes is generated using R2.11 (Bell Laboratories), and network analysis (CNA) of the correlation matrix is performed with the help of Cytoscape 2.7.0 software [123]. The correlation coefficient is defined as statistically significant at a *P*-value of less than 0.05.

### Electronic supplementary material

Below is the link to the electronic supplementary material.

**Supplementary Material 1: Table S1**. List of the 67 *S. cereale* GRAS genes identified in this study

**Supplementary Material 2: Table S2**. Analysis and distribution of conserved motifs in *Secale cereale* GRAS proteins

**Supplementary Material 3: Table S3**. The distribution of amino acid sites in the motifs in rye

**Supplementary Material 4: Table S4**. Analysis of Motif Enrichment in 67 GRAS proteins in rye

**Supplementary Material 5: Table S5**. Cis-regulatory elements in the promoter region of *ScGRAS* genes

**Supplementary Material 6: Table S6**. The tandem duplication events of *ScGRAS* genes in Weining

**Supplementary Material 7: Table S7**. The gene pair similarity comparison in EMBOSS Needle

**Supplementary Material 8: Table S8**. The three pairs of segmental duplicates in S. *cereale GRAS* genes

**Supplementary Material 9: Table S9**. One-to-one orthologous relationships between *Secale cereale* and *Arabidopsis* thaliana

**Supplementary Material 10: Table S10**. Results of Tajima’s D neutrality test

**Supplementary Material 11: Table S11**. Ka/Ks ratio distribution of gene pairs in different subfamilies in Weininng

**Supplementary Material 12: Table S12**. The relative expression levels of GRAS genes were detected at different stages of grain development under gibberellin treatment

**Supplementary Material 13: Table S13**. List of the 72 ScGRAS genes identified in Lo7

**Supplementary Material 14: Table S14**. One-to-one orthologous relationships between Lo7 and Weining

**Supplementary Material 15: Table S15**. Primer sequences for qPCR

**Supplementary Material 16: Figure S1**. Multiple sequence alignments of the *GRAS* domains of the members of 13 phylogenetic subfamilies of the *ScGRAS* protein family. The scheme at the top depicts the locations and boundaries of the LHR I, VHIID, LHR II, PFYRE, and SAW regions in the *GRAS* domain. **Figure S2**. Conserved sequence logo of GRAS proteins in rye. **Figure S3**. Conserved sequence logo in seven species. **Figure S4**. The correlations of 19 *S. cereale* GRAS genes in several plant organs. **Figure S5**. The correlations of 19 *S. cereale* GRAS genes during grain development. **Figure S6**. Gene expression of 19 *S. cereale GRAS* genes during six abiotic stresses (UV-A, flooding, PEG, NaCl, heat, and cold) at the seedling stage. The expression patterns of 19 *S. cereale GRAS* genes in leaf, root, and stem organs were examined via qRT-PCR. Error bars were obtained from three measurements. Lowercase letters above the bars indicate significant differences (? = 0.05, LSD) among the treatments. **Figure S7**. The correlations of 19 *S. cereale GRAS* genes in several abiotic stresses. **Figure S8**. Correlation network of the expression of *ScGRAS* family members in grains treated with different hormones. Among them, A, B, C and D are abscisic acid, gibberellin, auxin and paclobutrazol respectively. **Figure S9**. Unrooted phylogenetic tree showing relationships among GRAS genes of *S. cereale* (Weining and Lo7), *A. thaliana* and *O. sativa*. **Figure S10**. Synteny analyses of the GRAS genes between Weining and Lo7

## Data Availability

The entire *Secale cereale* genome sequence information was obtained from the NCBI (National Center for Biotechnology Information) GenBank website, the access number is JADQCU000000000. rye materials (Weining) used in the experiment were supplied by Prof. Kuiying Li of Anshun University. The datasets supporting the conclusions of this study are included in the article and its additional files.
