# Supplementary material for: GRAS gene family in rye (Secale cereale L.): genome-wide identification, phylogeny, evolutionary expansion and expression analyses
Source: BMC Plant Biol. 2024 Jan 13;24:46. doi: 10.1186/s12870-023-04674-1 (PMC10787399; doi:10.1186/s12870-023-04674-1)
Supplement: Supplementary file 16 — Supplementary Material 16: Figure S1. Multiple sequence alignments of the GRAS domains of the members of 13 phylogenetic subfamilies of the ScGRAS protein family. The scheme at the top depicts the locations and boundaries of the LHR I, VHIID, LHR II, PFYRE, and SAW regions in the GRAS domain. Figure S2. Conserved sequence logo of GRAS proteins in rye. Figure S3. Conserved sequence logo in seven species. Figure S4. The correlations of 19 S. cereale GRAS genes in several plant organs. Figure S5. The correlations of 19 S. cereale GRAS genes during grain development. Figure S6. Gene expression of 19 S. cereale GRAS genes during six abiotic stresses (UV-A, flooding, PEG, NaCl, heat, and cold) at the seedling stage. The expression patterns of 19 S. cereale GRAS genes in leaf, root, and stem organs were examined via qRT-PCR. Error bars were obtained from three measurements. Lowercase letters above the bars indicate significant differences (? = 0.05, LSD) among the treatments. Figure S7. The correlations of 19 S. cereale GRAS genes in several abiotic stresses. Figure S8. Correlation network of the expression of ScGRAS family members in grains treated with different hormones. Among them, A, B, C and D are abscisic acid, gibberellin, auxin and paclobutrazol respectively. Figure S9. Unrooted phylogenetic tree showing relationships among GRAS genes of S. cereale (Weining and Lo7), A. thaliana and O. sativa. Figure S10. Synteny analyses of the GRAS genes between Weining and Lo7 [file 12870_2023_4674_MOESM16_ESM.docx]

**Supplementary Materials**

**
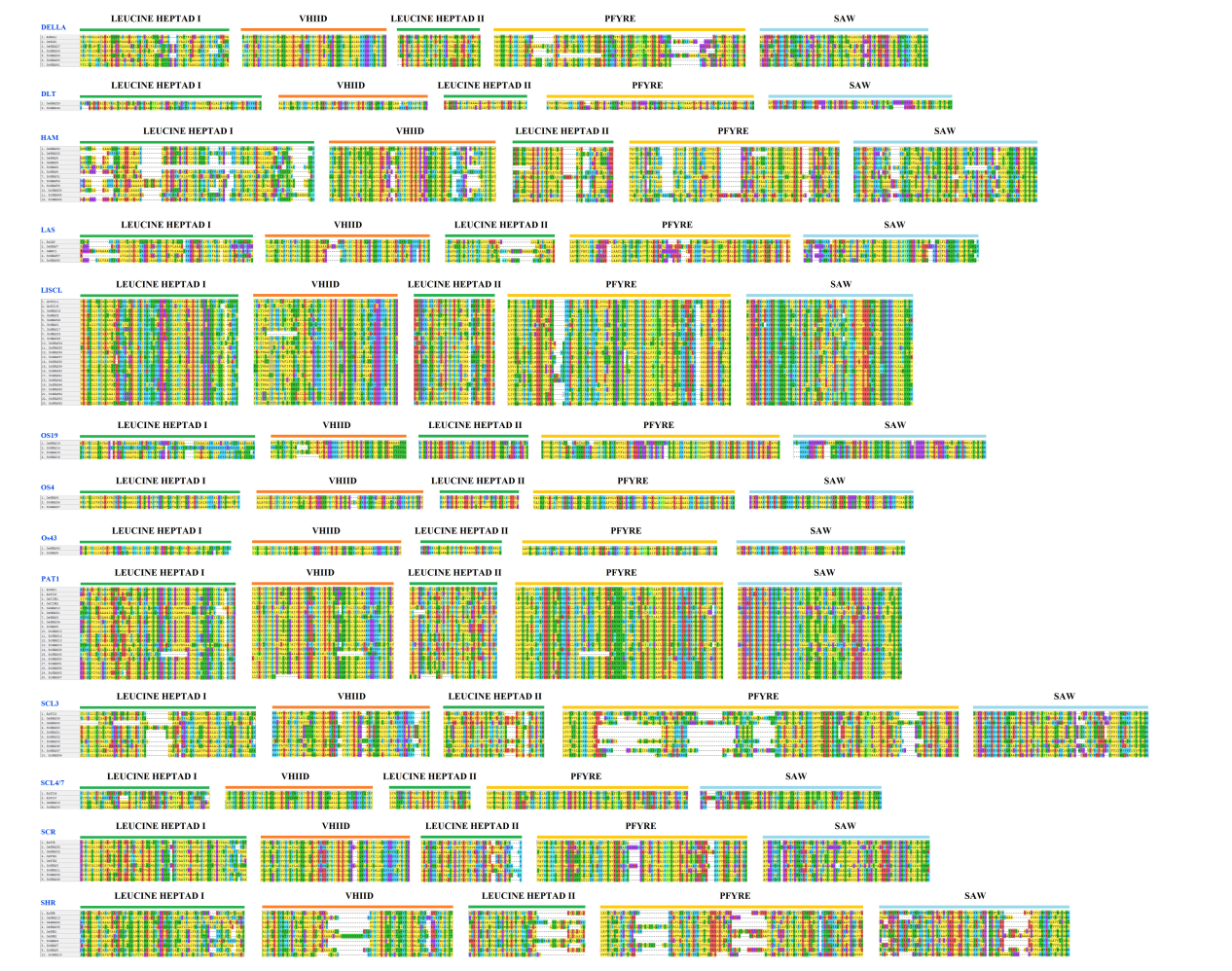
**

**Figure S1.** Multiple sequence alignments of the *GRAS* domains of the members of 13 phylogenetic subfamilies of the *ScGRAS* protein family. The scheme at the top depicts the locations and boundaries of the LHR I, VHIID, LHR II, PFYRE, and SAW regions in the *GRAS* domain.


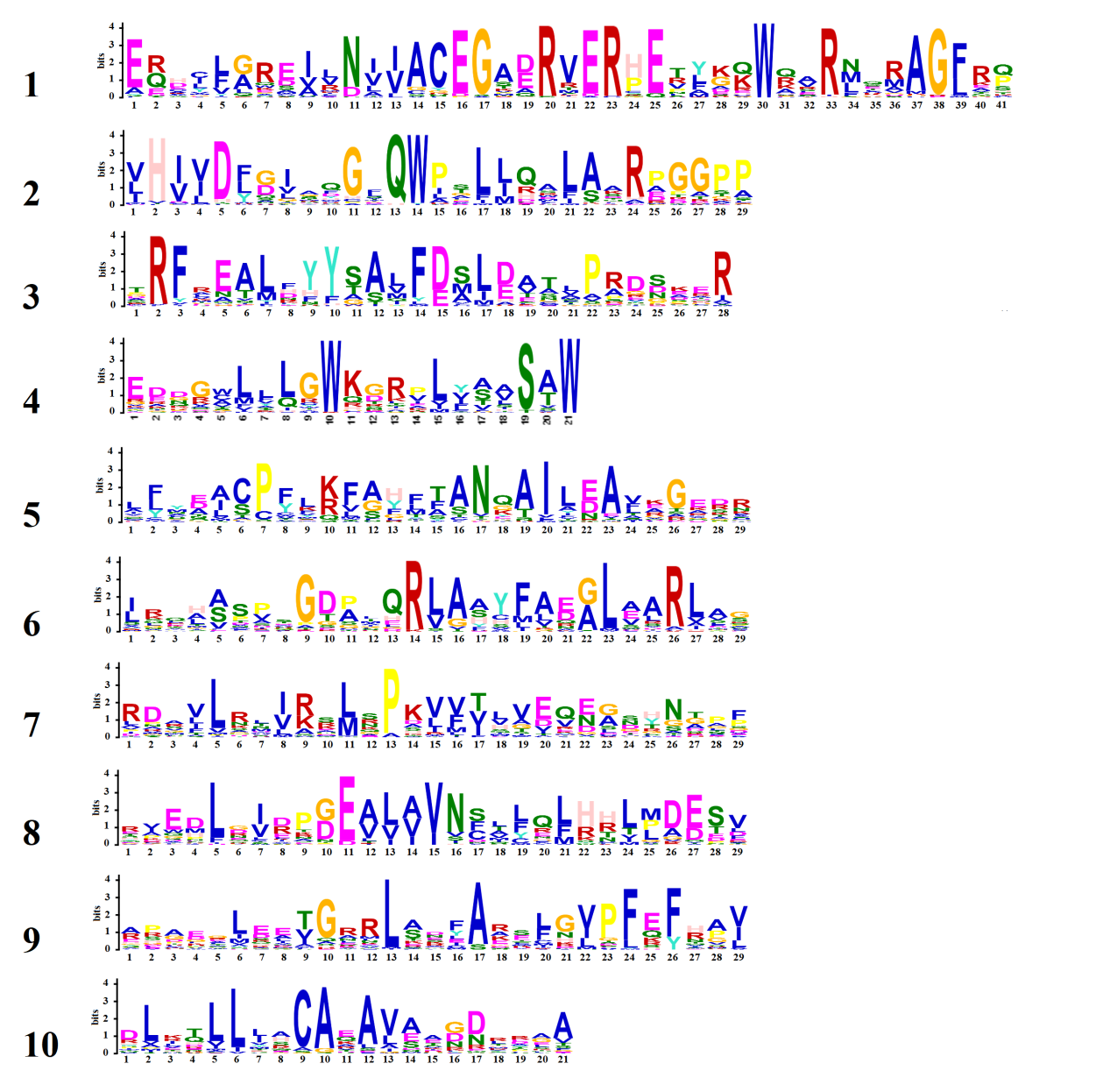


**Figure S2.** Conserved sequence logo of GRAS proteins in rye.


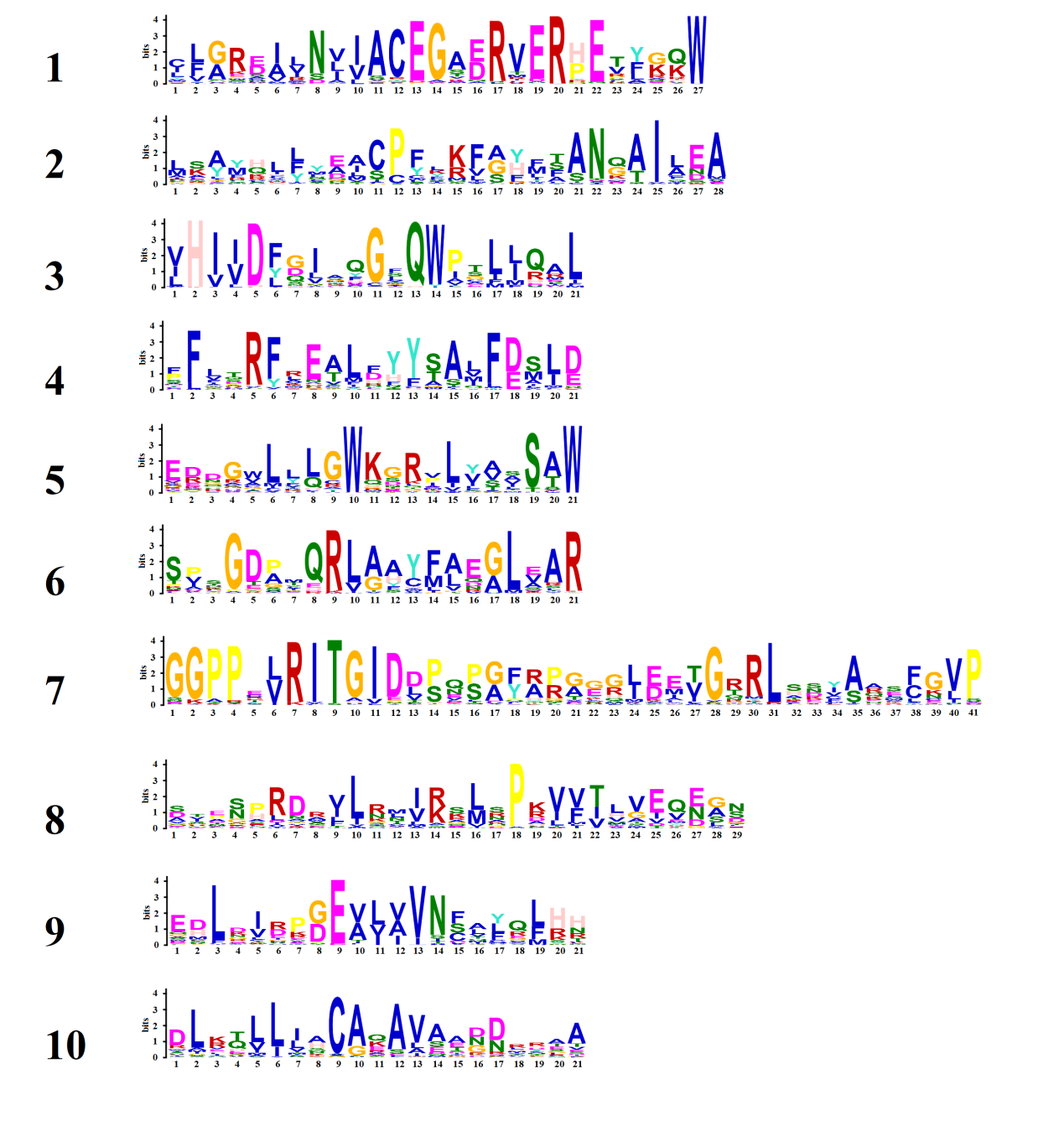


**Figure S3.** Conserved sequence logo in seven species.


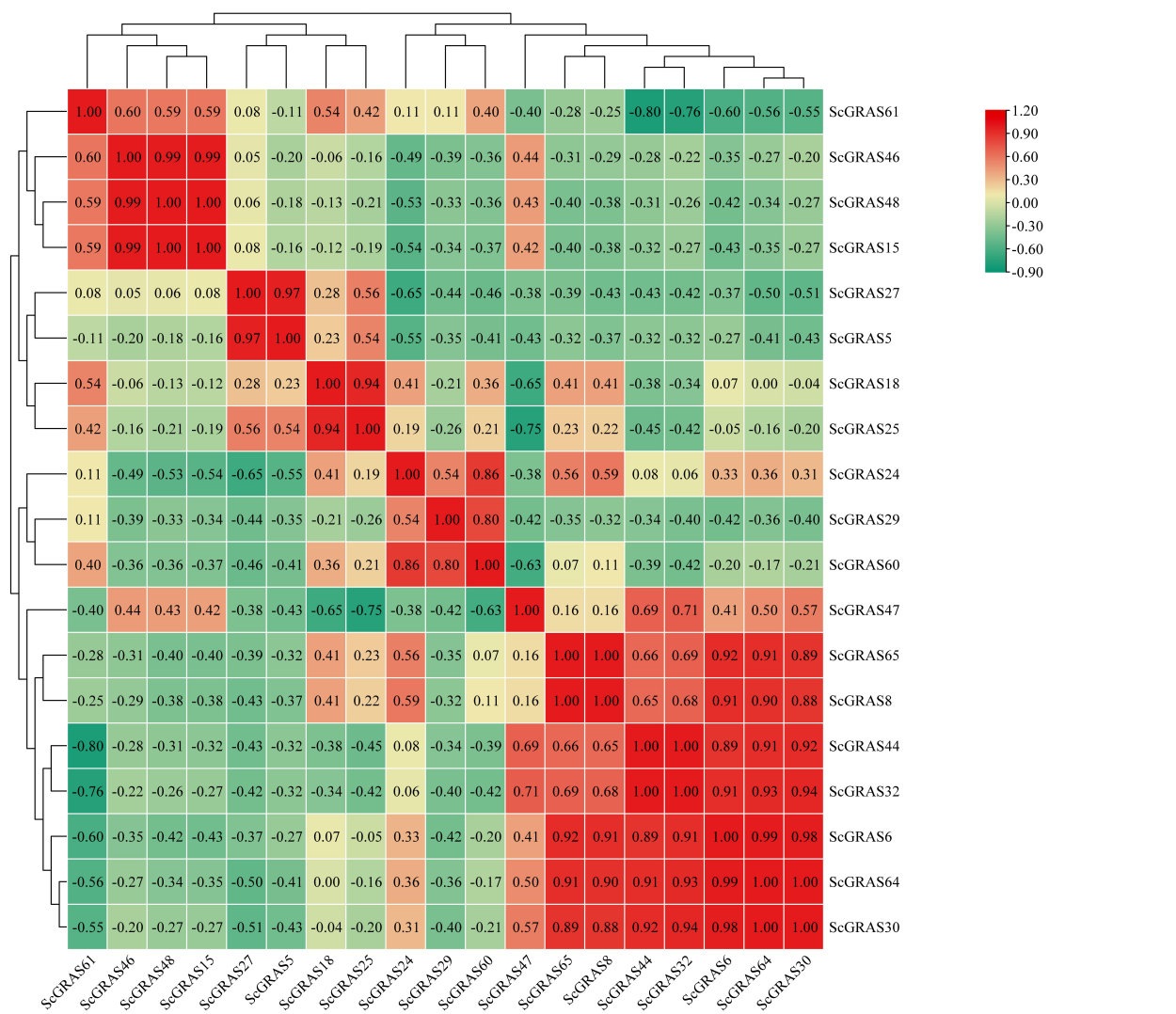


**Figure S4.** The correlations of 19 *S. cereale GRAS* genes in several plant organs.


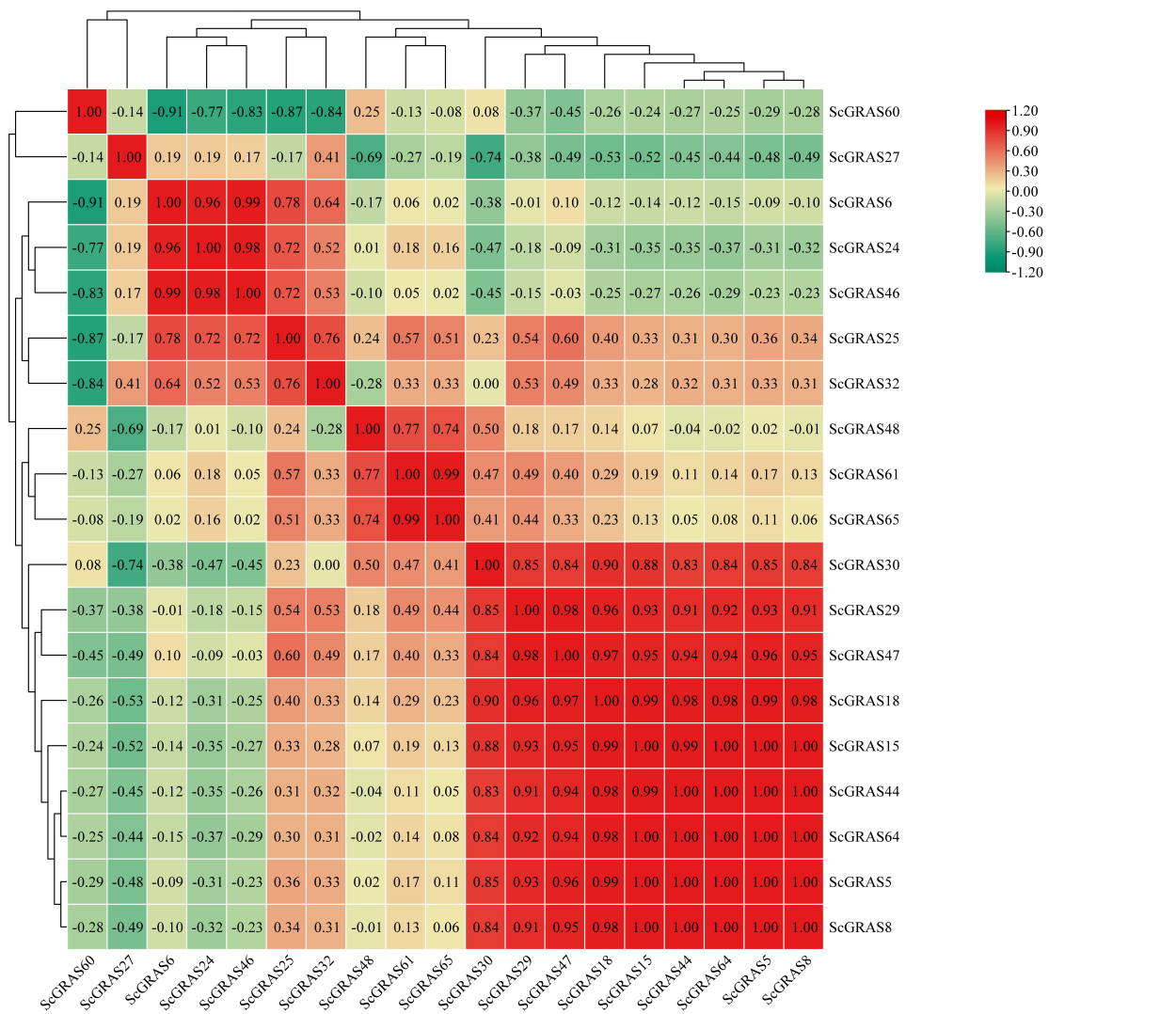


**Figure S5.** The correlations of 19 *S. cereale GRAS* genes during grain development.


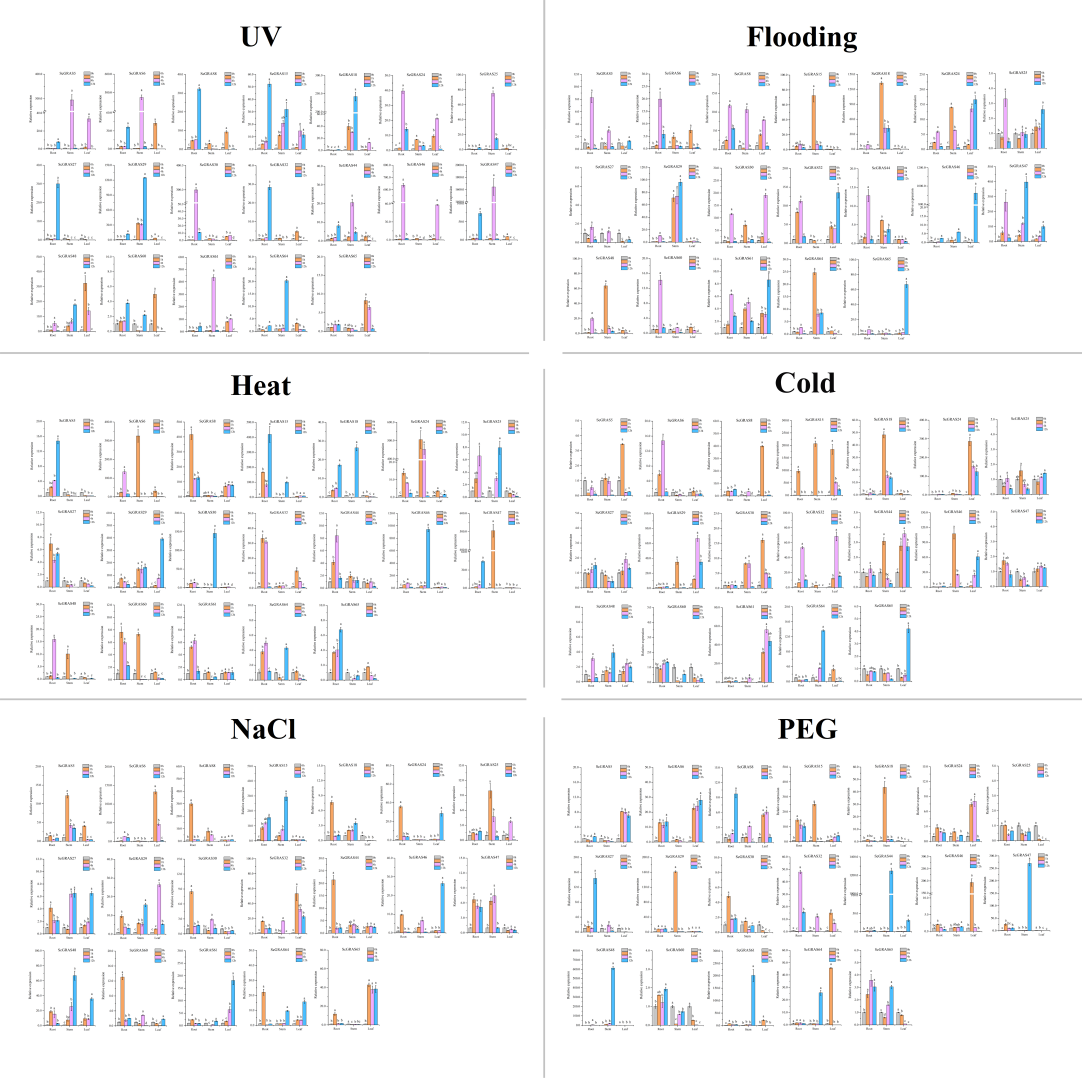


**Figure S6.** Gene expression of 19 *S. cereale GRAS* genes during six abiotic stresses (UV-A, flooding, PEG, NaCl, heat, and cold) at the seedling stage. The expression patterns of 19 *S. cereale GRAS* genes in leaf, root, and stem organs were examined via qRT-PCR. Error bars were obtained from three measurements. Lowercase letters above the bars indicate significant differences (α = 0.05, LSD) among the treatments.


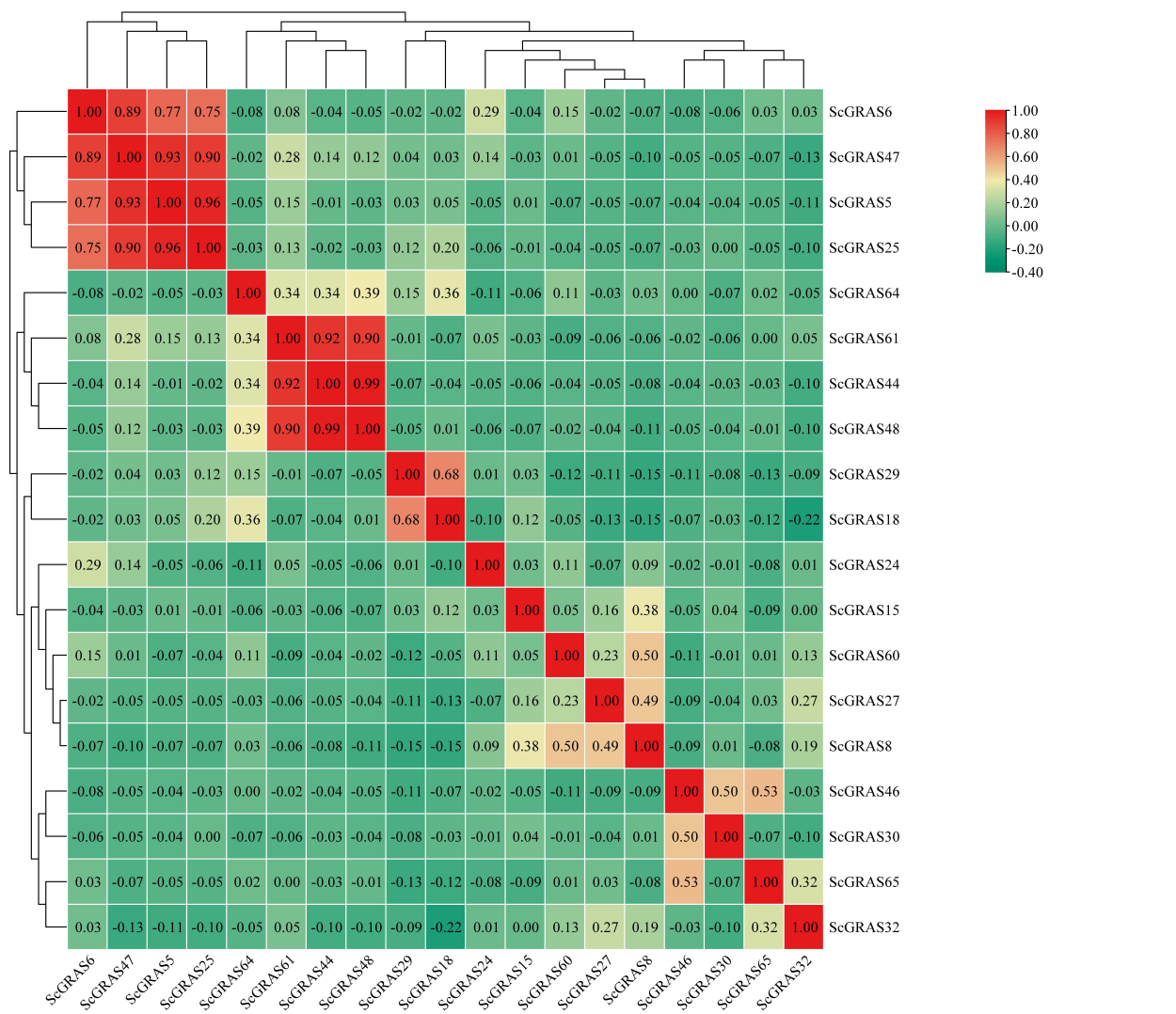


**Figure S7.** The correlations of 19 *S. cereale GRAS* genes in several abiotic stresses.


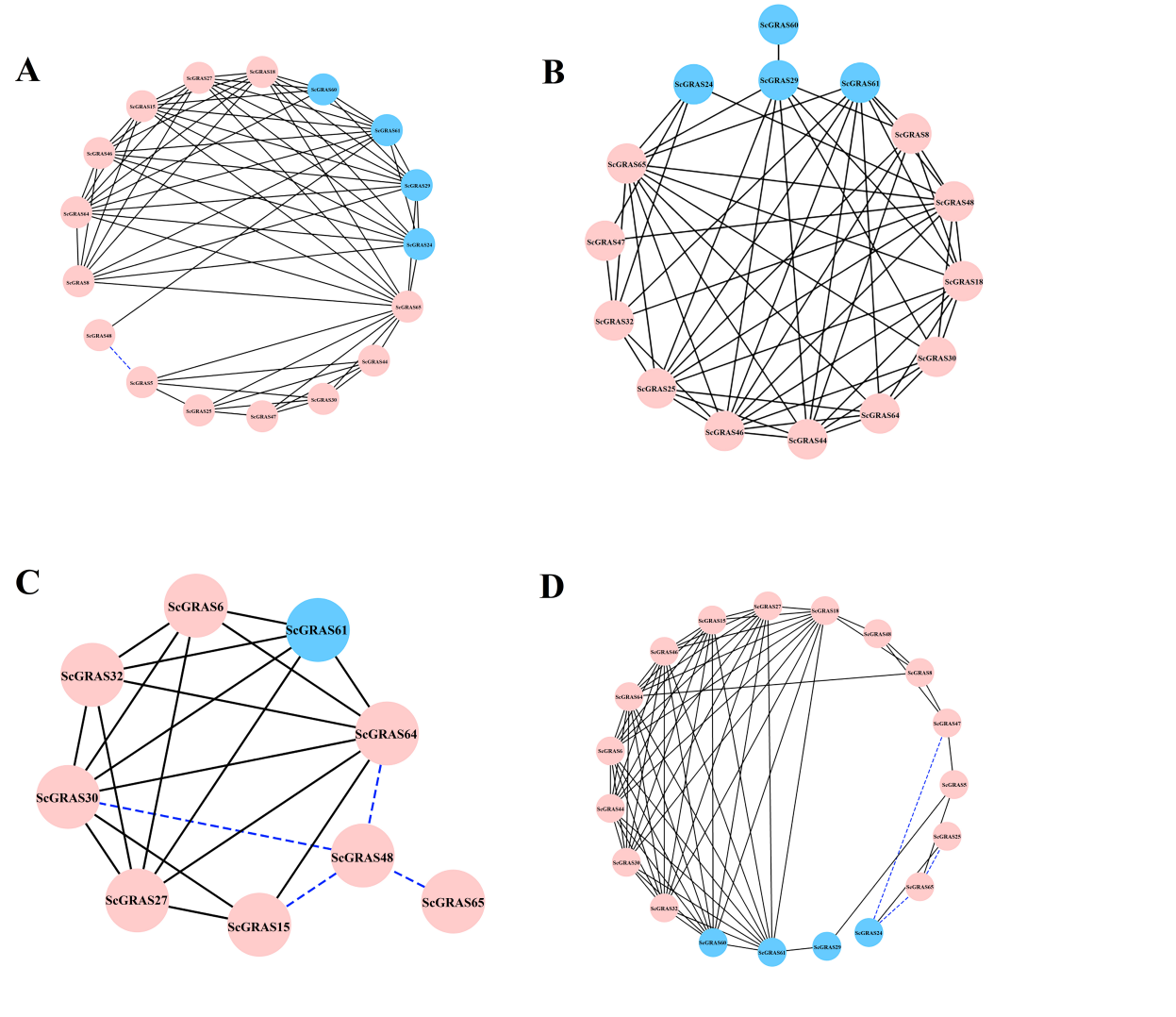


**Figure S8.** Correlation network of the expression of *ScGRAS* family members in grains treated with different hormones. Among them, A, B, C and D are abscisic acid, gibberellin, auxin and paclobutrazol respectively.

**
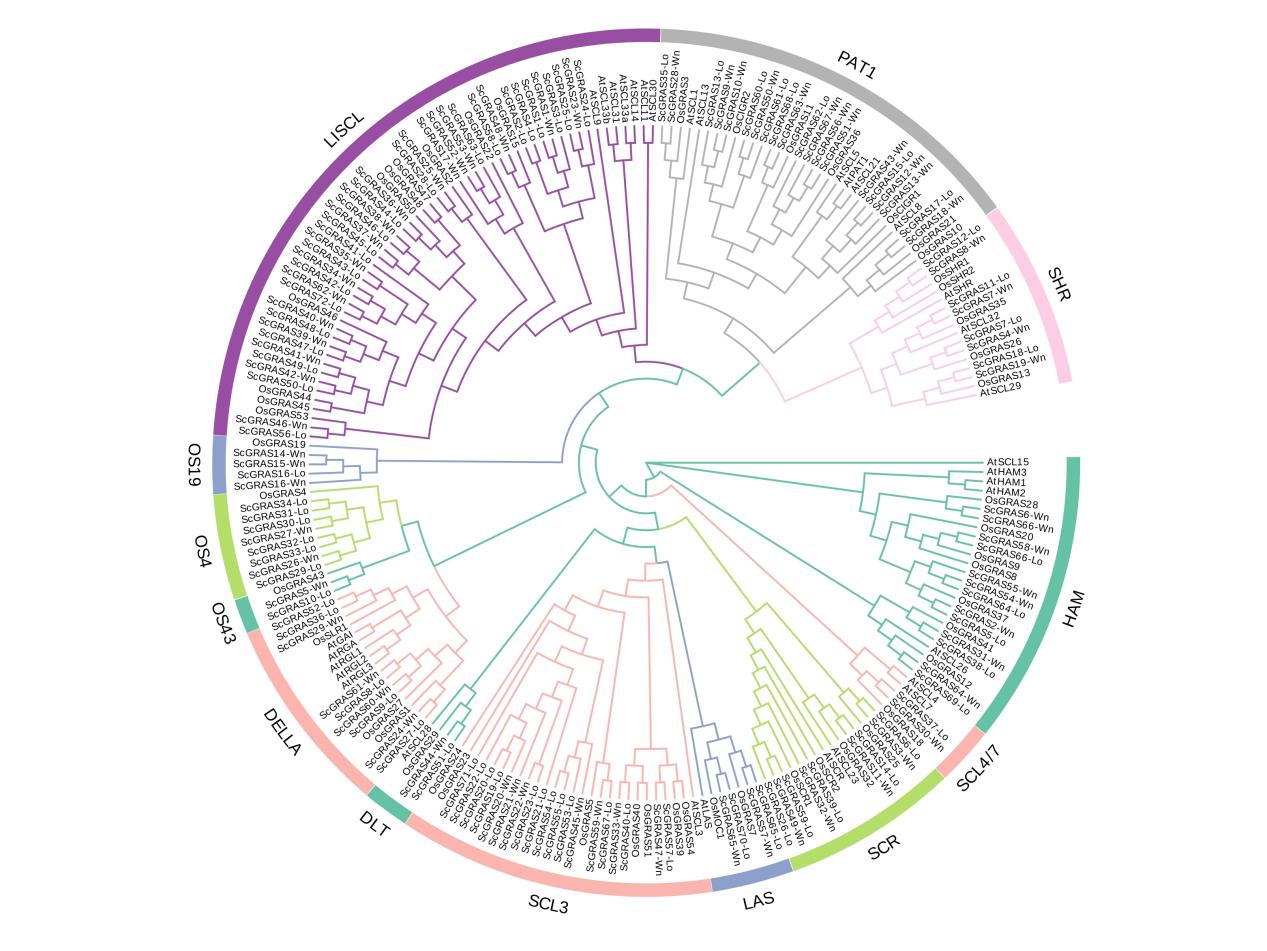
**

**Figure S9.** Unrooted phylogenetic tree showing relationships among GRAS genes of *S. cereale* (Weining and Lo7), *A. thaliana* and *O. sativa*.


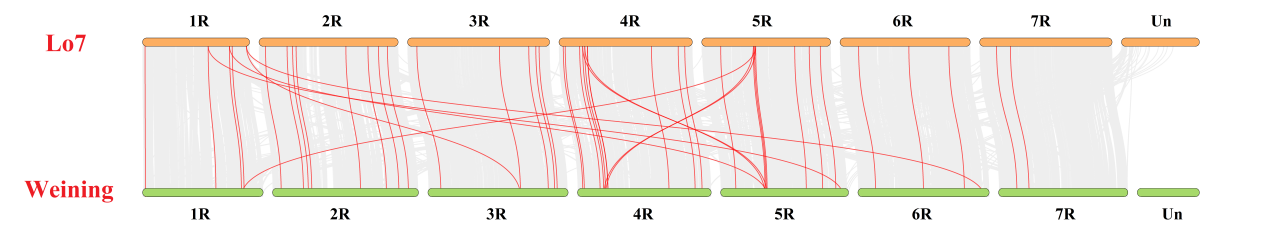


**Figure S10.** Synteny analyses of the GRAS genes between Weining and Lo7.
